# Association of metabolomic aging acceleration and body mass index phenotypes with mortality and obesity‐related morbidities

**DOI:** 10.1111/acel.14435

**Published:** 2024-12-12

**Authors:** Xiaomin Zeng, Ruiye Chen, Danli Shi, Xiayin Zhang, Ting Su, Yaxin Wang, Yijun Hu, Mingguang He, Honghua Yu, Xianwen Shang

**Affiliations:** ^1^ Guangdong Eye Institute, Department of Ophthalmology, Guangdong Provincial People's Hospital (Guangdong Academy of Medical Sciences) Southern Medical University Guangzhou China; ^2^ The Ophthalmic Epidemiology Department Centre for Eye Research Australia Melbourne Victoria Australia; ^3^ Department of Surgery University of Melbourne Melbourne Victoria Australia; ^4^ School of Optometry The Hong Kong Polytechnic University Kowloon Hong Kong; ^5^ Research Centre for SHARP Vision (RCSV) The Hong Kong Polytechnic University Kowloon Hong Kong; ^6^ Centre for Eye and Vision Research (CEVR) Hong Kong Hong Kong; ^7^ Guangdong Provincial Key Laboratory of Artificial Intelligence in Medical Image Analysis and Application Guangzhou China

**Keywords:** body mass index, metabolomic aging acceleration, mortality, obesity‐related morbidities, risk stratification

## Abstract

This study aims to investigate the association between metabolomic aging acceleration and body mass index (BMI) phenotypes with mortality and obesity‐related morbidities (ORMs). 85,458 participants were included from the UK Biobank. Metabolomic age was determined using 168 metabolites. The Chronological Age‐Adjusted Gap was used to define metabolomically younger (MY) or older (MO) status. BMI categories were defined as normal weight, overweight, and obese. Participants were categorized into MY normal weight (MY‐NW, reference), MY overweight (MY‐OW), MY obesity (MY‐OB), MO normal weight (MO‐NW), MO overweight (MO‐OW), and MO obesity (MO‐OB). Mortality and 43 ORMs were identified through death registries and hospitalization records. Compared with MY‐NW phenotype, MO‐OB phenotype yielded increased risk of mortality and 32 ORMs, followed by MO‐OW with mortality and 27 ORMs, MY‐OB with mortality and 26 ORMs, MY‐OW with 21 ORMs, and MO‐NW with mortality and 14 ORMs. Consistently, MO‐OB phenotype showed the highest risk of developing obesity‐related multimorbidities, followed by MY‐OB phenotype, MO‐OW phenotype, MY‐OW phenotype, and MO‐NW phenotype. Additive interactions were found between metabolomic aging acceleration and obesity on CVD‐specific mortality and 10 ORMs. Additionally, individuals with metabolomic aging acceleration had higher mortality and cardiovascular risk, even within the same BMI category. These findings suggest that metabolomic aging acceleration could help stratify mortality and ORMs risk across different BMI categories. Weight management should also be extended to individuals with overweight or obesity even in the absence of accelerated metabolomic aging, as they face increased healthy risk compared with MY‐NW individuals. Additionally, delaying metabolic aging acceleration is needed for all metabolomically older groups, including those with normal weight

## Graphical abstract

Metabolomic aging acceleration may help stratify mortality and obesity‐related morbidities risk across different BMI categories. Weight management should address Individuals with overweight or obesity, regardless of metabolomic aging, while delaying metabolomic aging is crucial for all metabolomically older groups, including those with normal weight.

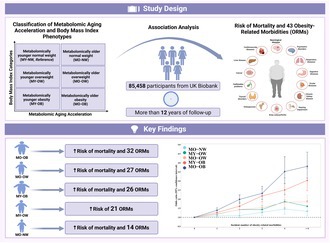

**Abbreviations**
BMI: body mass index
CAAG: chronological age‐adjusted age gap
CI: confidence intervals
CKD: chronic kidney disease
COPD: chronic obstructive pulmonary disease
CVD: cardiovascular diseases
FDR: false discovery rate
GERD: gastroesophageal reflux disease
GRS: genetic risk score
HR: hazard ratio
ICD: International Classification of Diseases
INFLA‐score: low‐grade inflammation score
MAA: metabolomic aging acceleration
MAA‐BMI: metabolomic aging acceleration and body mass index
MO: metabolomically older
MO‐NW: metabolomically older normal weight
MO‐OB: metabolomically older obesity
MO‐OW: metabolomically older overweight
MY: metabolomically younger
MY‐NW: metabolomically younger normal weight
MY‐OB: metabolomically younger obesity
MY‐OW: metabolomically younger overweight
NAFLD: nonalcoholic fatty liver disease
NHS: National Health Service
ORMs: obesity‐related morbidities
SD: standard deviation
TDI: Townsend deprivation index

## 1 INTRODUCTION

The global public health landscape is currently facing an unprecedented challenge from overweight and obesity. The World Health Organization reported that 2.5 billion adults were overweight, and 890 million of these were living with obesity in 2022, accounting for 43% and 16% of adults worldwide, respectively (World Health Organization, 2020). Overweight and obesity not only pose a high risk of mortality and a range of obesity‐related morbidities (ORMs), including cardiovascular diseases (CVD), cancers, chronic respiratory diseases, digestive disorders, and adverse mental health (Murray et al., 2020), but also contribute significantly to the economic burden across countries due to the treatment of obesity and its complications (Okunogbe et al., 2022). In this context, it is essential to prioritize patients who would benefit most from weight loss interventions to reduce treatment costs, which requires tools to measure mortality and ORMs risk in individuals with overweight and obesity (Blüher, 2020).

Current guidelines for obesity treatment mainly focus on individuals with a body mass index (BMI) of 30 kg/m^2^ or higher (Garvey et al., 2016; Yumuk et al., 2015); however, there is significant heterogeneity in health risk for individuals even with the same BMI category. For instance, some individuals who are overweight or obese are reported to have a lower or comparable risk of mortality compared with those who are of normal weight (Flegal et al., 2013; Zembic et al., 2021). In light of this, establishing a risk classification for individuals in different BMI categories is attracting considerable attention (Stefan & Schulze, 2023). Obesity‐related metabolic dysfunction has major clinical and public health implications (Beyene et al., 2023). A growing body of literature has revealed that metabolic status might allow for better stratification of individuals with overweight or obesity (Stefan et al., 2013). Recently, metabolomic age, a predicted age derived from blood metabolomic profiling, has been verified to reflect overall health status, particularly metabolic aging rates (Lau et al., 2024; Shang et al., 2024). Considering that ORMs are associated with accelerated cellular processes observed in normal aging, and that both obesity and accelerated aging can predispose individuals to chronic health complications (Brito et al., 2022; Santos & Sinha, 2021), introducing metabolomic aging may offer pathophysiological insights into the heterogeneity in different BMI categories (Wei et al., 2023).

In this article, we hypothesized that metabolomic aging acceleration could serve as a novel tool to stratify mortality and ORM risks across different BMI groups. Therefore, the primary aim of this study is to investigate the association of metabolomic aging acceleration and BMI phenotypes with the risk of mortality and ORMs using data from the UK Biobank. In addition, given the role of inflammation in the pathways linking obesity to health conditions, we further investigate the mediating effect of chronic inflammation on the relationship between metabolomically younger BMI phenotypes and risk of health outcomes.

## 2 METHODS

### 2.1 Study population

This analysis utilized data from the UK Biobank, a population‐based prospective cohort study. In brief, approximately 9.2 million people aged 40–73 who were registered with the National Health Service (NHS) were invited to participate. Over 500,000 participants were assessed at baseline between 2006 and 2010 across 22 assessment centers in the UK. Participants provided data on geographic factors, lifestyle, and other health‐related aspects through comprehensive baseline questionnaires, interviews, and physical measurements.

In this study, we obtained data from 502,367 participants of the UK Biobank. After excluding those with missing metabolomics data (*n* = 391,675), missing body measurements (*n* = 997), missing other relevant covariates (*n* = 23,996), or lost to follow‐up (*n* = 241), a total of 85,458 participants were included in the final analysis (Figure S1). The study was ethically approved by the National Information Governance Board for Health and Social Care and the NHS North West Multicenter Research Ethics Committee (reference 11/NW/0382). All participants provided informed consent during the baseline assessment. The study adhered to the principles of the Declaration of Helsinki and was conducted under UK Biobank application number 101032.

### 2.2 Ascertainment of exposures

The methods of metabolomic age prediction using machine learning were described in detail in our previous study (Shang et al., 2024). Briefly, multiple linear regression models with chronological age as the dependent variable were used to develop metabolomic age based on 168 metabolites. The metabolomic age gap was defined as the deviation between the metabolomic age and the chronological age. Given the age gap for individuals with different ages might represent different metabolomic aging levels, the chronological age‐adjusted age gap (CAAG) was calculated as a residual of the metabolomic age gap adjusted for chronological age by the linear regression model (Yang et al., 2022). Participants with a CAAG greater than 0 were classified as having metabolomic aging acceleration (MAA) and were defined as metabolomically older (MO), while those with a CAAG less than 0 were classified as not having metabolomic aging acceleration and were defined as metabolomically younger (MY).

BMI categories included normal weight (BMI < 25 kg/m^2^), overweight (25 ≤ BMI < 30 kg/m^2^), and obesity (BMI ≥ 30 kg/m^2^) (Grundy et al., 2005). Participants were categorized into the following six MAA and BMI (MAA‐BMI) phenotypes: metabolomically younger normal weight (MY‐NW, reference group), metabolomically younger overweight (MY‐OW), metabolomically younger obesity (MY‐OB), metabolomically older normal weight (MO‐NW), metabolomically older overweight (MO‐OW), and metabolomically older obesity (MO‐OB).

### 2.3 Ascertainment of outcomes

The outcomes included mortality (all‐cause, CVD‐specific, and cancer‐specific) and 43 ORMs (Kim et al., 2024), including CVDs, cancers, neurological diseases, digestive diseases, respiratory diseases, mental diseases, ocular diseases, thyroid disorders, chronic kidney disease (CKD), osteoporosis, and so on. Baseline diseases were defined if participants reported that they had ever been told by a doctor that they had the diseases (Table S6). Incident cases were identified using International Classification of Diseases (ICD) 10 and ICD 9 (Table S5) through inpatient hospital records and mortality registers. The follow‐up time was calculated from the date of baseline assessment to the date of disease onset, death, or the end of follow‐up (30th September 2021), whichever came first. Moreover, to evaluate the risk of obesity‐related multimorbidity, we quantified the number of incident cases of the 43 ORMs that occurred during the follow‐up period for participants who were free from these diseases at baseline. Participants were classified into five groups based on the number of new ORMs occurrences during follow‐up: 0, 1, 2, 3, 4, and ≥5.

### 2.4 Ascertainment of Medicator

The low‐grade inflammation score (INFLA‐score), a well‐established scoring system for chronic low‐grade inflammation, is computed based on 10 tiles of C‐reactive protein, white blood cell count, platelet count, and the neutrophil‐to‐lymphocyte ratio. Biomarker levels in the highest deciles (7th to 10th) are assigned values from +1 to +4, while those in the lowest deciles (1st to 4th) are assigned values from −4 to −1. The INFLA‐score is the arithmetic sum of these scores, ranging from −16 to +16, where a higher score indicates a greater level of low‐grade inflammation (Pounis et al., 2016; Shi et al., 2022).

### 2.5 Ascertainment of covariates

Covariates included baseline age, sex (female or male), Townsend deprivation index (TDI), ethnicity (white or non‐white), education attainment (≥college degree or <college degree), smoking status (never, previous, or current), and drinking status (never, previous, or current). Besides, physical activity was evaluated using International Physical Activity Questionnaire and recorded as summed mins of performing walking, moderate, and vigorous activity per week. Healthy diet was adapted from the American Heart Association Guidelines (Rutten‐Jacobs et al., 2018). Sleep duration was assessed based on the question, “About how many hours' sleep do you get in every 24 hours?” (Lloyd‐Jones et al., 2022). Longevity genetic risk score (GRS) was computed using 78 single‐nucleotide polymorphisms with a higher score representing longer potential longevity.

### 2.6 Statistical analysis

Descriptive statistics reported baseline characteristics of participants, with continuous variables reported as mean ± standard deviation (SD) and categorical variables summarized as frequency (percentage). Differences in baseline characteristics by MAA‐BMI phenotypes were tested using ANOVA tests for continuous variables and the Chi‐square test for categorical variables. Cox proportional hazard regression was used to estimate the hazard ratios (HRs) and 95% confidence intervals (CI) of six MAA‐BMI phenotypes with risk of mortality and 43 ORMs. The proportion attributable to interaction, representing the measure of additive interaction between metabolomic aging acceleration (younger vs. older) and obesity status (normal weight vs. obesity), was considered statistically significant if the 95% CI did not include 0. We examined two models: Model 1 was adjusted for age and sex; Model 2 was additionally adjusted for ethnicity, TDI, educational attainment, physical activity, diet, sleep duration, smoking, drinking, and longevity GRS. Prostate cancer was analyzed only in males. Breast cancer was analyzed only in females. Furthermore, multinomial logistic regression was used to estimate the association of MAA‐BMI phenotypes with the incident number of ORMs, adjusting for covariates in Model 2. We then estimated the effect of metabolomic aging acceleration on risk of mortality and ORMs in different BMI categories. Multiplicative interaction was evaluated by including the product term of metabolomic aging acceleration (younger or older) and BMI (continuous) in the model. Next, mediation analyses were conducted to examine the role of the INFLA‐score in the association between metabolomically younger phenotypes of overweight/obesity and the risk of outcomes (MY‐NW was set as the reference group). Finally, we conducted three sensitivity analyses: first, we excluded individuals who developed the corresponding disease within 2 years of follow‐up to mitigate potential reverse causation. Second, we randomly selected 50% of the population to further validate the reliability of our findings. Third, we performed sex‐stratified and age‐stratified analyses to assess whether the association between MAA‐BMI phenotypes and the risk of mortality and ORMs was modified by sex and age. Data analyses were performed using Stata (version 17.0) and R (version 4.3.3), with *p*‐values being two‐tailed and a significance level set at <0.05. The Benjamini‐Hochberg procedure was applied to control for false discovery rate (FDR) at the 5% level (Benjamini & Hochberg, 1995).

## 3 RESULTS

### 3.1 Baseline characteristics

Table 1 summarizes the baseline characteristics of participants stratified by six MAA‐BMI phenotypes. Among the 85,458 participants, 19.00% were classified as MY‐NW, 14.24% as MO‐NW, 22.25% as MY‐OW, 21.05% as MO‐OW, 9.16% as MY‐OB, and 14.31% as MO‐OB. Compared with MY‐NW individuals, those with other phenotypes tended to be older, with higher metabolomic age, CAAG, and BMI, as well as lower educational attainment and worse socioeconomic status. Participants with overweight and obesity were also more likely to be male, smokers, have lower physical activity, shorter sleep duration, and unhealthier diet.

**TABLE 1:** Baseline characteristics stratified by metabolomic aging acceleration and body mass index phenotypes.

Baseline characteristics	MY‐NW	MO‐NW	MY‐OW	MO‐OW	MY‐OB	MO‐OB	*p*
Number of patients (%)	16,238 (19.00)	12,165 (14.24)	19,014 (22.25)	17,985 (21.05)	7831 (9.16)	12,225 (14.31)	–
Age, years, mean (SD)	55.21 (8.30)	55.75 (8.15)	56.85 (8.08)	56.73 (8.10)	56.91 (7.82)	56.30 (7.95)	<0.001
Metabolomic age, years, mean (SD)	53.14 (3.26)	58.97 (3.19)	53.85 (3.12)	59.36 (3.14)	54.14 (3.03)	59.62 (3.18)	<0.001
Chronological age‐adjusted age gap, years, mean (SD)	−0.84 (0.62)	0.72 (0.61)	−0.77 (0.61)	0.76 (0.61)	−0.69 (0.60)	0.86 (0.66)	<0.001
Sex, *n* (%)							
Female	9906 (61.01)	8050 (66.17)	7739 (40.70)	8644 (48.06)	3484 (44.49)	6351 (51.95)	<0.001
Male	6332 (38.99)	4115 (33.83)	11,275 (59.30)	9341 (51.94)	4347 (55.51)	5874 (48.05)	
Ethnicity, *n* (%)							
White	15,455 (95.18)	11,669 (95.92)	18,113 (95.26)	17,129 (95.24)	7405 (94.56)	11,542 (94.41)	<0.001
Non‐white	783 (4.82)	496 (4.08)	901 (4.74)	856 (4.76)	426 (5.44)	683 (5.59)	
Educational attainment, *n* (%)							
≥College degree	6995 (43.08)	5103 (41.95)	6422 (33.78)	6082 (33.82)	2096 (26.77)	3426 (28.02)	<0.001
<College degree	9243 (56.92)	7062 (58.05)	12,592 (66.22)	11,903 (66.18)	5735 (73.23)	8799 (71.98)	
TDI, mean ± SD	−1.61 (2.93)	−1.59 (2.98)	−1.59 (2.93)	−1.52 (2.99)	−1.08 (3.14)	−0.94 (3.25)	<0.001
Body size mass, kg/m^2^	22.82 (1.55)	22.93 (1.50)	27.22 (1.39)	27.36 (1.41)	33.20 (3.17)	34.26 (4.11)	<0.001
Physical activity (MET‐minutes/week)	2804.91 (2707.46)	2850.63 (2713.10)	2740.34 (2789.86)	2647.42 (2688.05)	2470.95 (2746.30)	2221.70 (2534.83)	<0.001
Healthy diet, *n* (%)	7234 (44.55)	6313 (51.89)	7525 (39.58)	8397 (46.69)	2756 (35.19)	5123 (41.91)	<0.001
Sleep duration (hours)	7.18 (1.02)	7.20 (1.07)	7.15 (1.09)	7.18 (1.13)	7.08 (1.24)	7.10 (1.28)	<0.001
Smoking, *n* (%)							
Never	9708 (59.79)	7061 (58.04)	10,289 (54.11)	9553 (53.12)	3941 (50.33)	6215 (50.84)	<0.001
Former	4814 (29.65)	3763 (30.93)	6907 (36.33)	6591 (36.65)	3125 (39.91)	4842 (39.61)	
Current	1716 (10.57)	1341 (11.02)	1818 (9.56)	1841 (10.24)	765 (9.77)	1168 (9.55)	
Drinking, *n* (%)							
Never	579 (3.57)	410 (3.37)	631 (3.32)	637 (3.54)	358 (4.57)	622 (5.09)	<0.001
Former	527 (3.25)	338 (2.78)	599 (3.15)	579 (3.22)	304 (3.88)	562 (4.60)	
Current	15,132 (93.19)	11,417 (93.85)	17,784 (93.53)	16,769 (93.24)	7169 (91.55)	11,041 (90.31)	
Longevity GRS, mean (SD)^a^	0.49 (0.05)	0.49 (0.05)	0.49 (0.05)	0.49 (0.05)	0.49 (0.05)	0.49 (0.05)	0.801

*Note*: Data are presented using means (SD), or numbers (percentage). One‐way ANOVA was used to test the difference of continuous variables across subgroups and *χ* ^2^ for categorical variables.

Abbreviations: GRS, genetic risk scores ; MO‐NW, metabolomically older‐normal weight; MO‐OW, metabolomically older‐overweight; MO‐OB, metabolomically older‐obesity; MY‐NW, metabolomically younger‐normal weight; MY‐OW, metabolomically younger‐overweight; MY‐OB, metabolomically younger‐obesity; SD, standard deviation; TDI, Townsend deprivation index.

^a^ Longevity GRS was calculated for longevity was calculated using 78 single‐nucleotide polymorphisms.

### 3.2 Association of MAA‐BMI phenotypes with risk of mortality and ORMs

**FIGURE 1:**
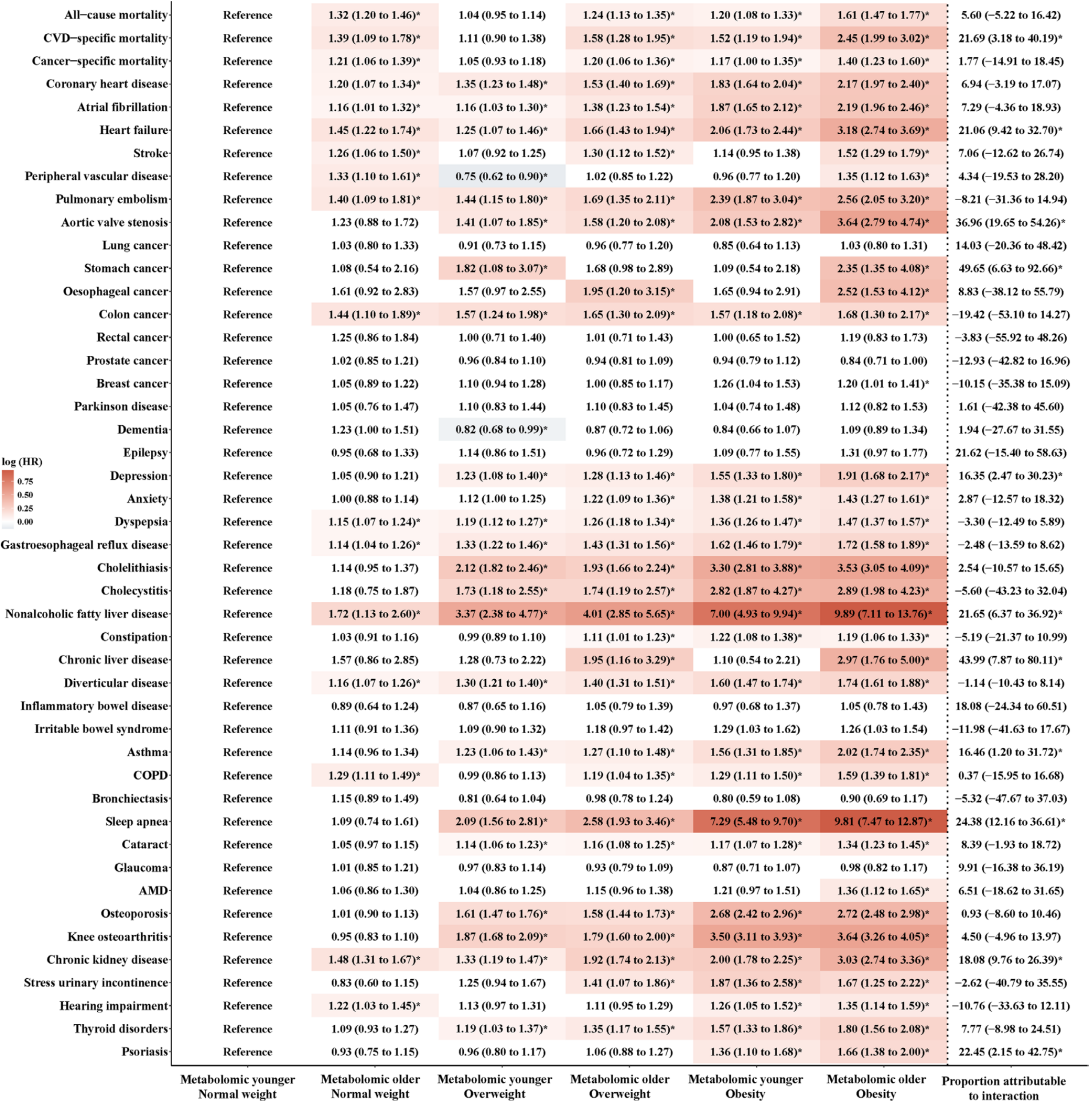
Association of metabolomic aging acceleration and body mass index phenotypes with risk of mortality and obesity‐related morbidities. The cox proportional hazard regression was used to estimate the association of metabolomic aging acceleration and body mass index phenotypes with risk of mortality and obesity‐related morbidities, adjusting for age, sex, ethnicity, Townsend deprivation index, educational attainment, physical activity, healthy diet, sleep duration, smoking, drinking, and longevity genetic risk scores. Metabolomic younger normal weight was set as the reference group. Prostate cancer was analyzed only in males. Breast cancer was analyzed only in females. Proportion attributable due to interaction and corresponding 95% CI was used as the measure of additive interaction between the metabolomic aging acceleration (younger vs. older) and obesity status (normal weight vs. obesity), and the additive interaction was statistically significant when its confidence interval did not include 0. The asterisk (*) indicates a significant association through two‐sided statistical tests. AMD, age‐related macular degeneration; COPD, chronic obstructive pulmonary disease; CVD, cardiovascular disease; HR, hazard ratio.

The follow‐up duration and incident cases varied across mortality and different ORMs (Table S1). The median follow‐up duration ranged from 12.44 years for dyspepsia to 12.61 years for mortality. The number of newly diagnosed cases ranged from 186 for stomach cancer to 10,174 for dyspepsia. Compared with MY‐NW group, individuals with MO‐OB had increased risk of mortality and 32 ORMs after adjusting for covariates in model 2 and controlling for FDR, followed by MO‐OW with mortality and 27 ORMs, MY‐OB with mortality and 26 ORMs, MY‐OW with 21 ORMs, and MO‐NW with mortality and 14 ORMs. Additionally, individuals with MO‐OB exhibited the highest HRs for mortality and most ORMs (30 out of 43). Additive interaction was observed between the metabolomic aging acceleration and obesity on CVD‐specific mortality and 10 ORMs, including heart failure (HF), aortic valve stenosis, stomach cancer, depression, nonalcoholic fatty liver disease (NAFLD), chronic liver disease, asthma, sleep apnea, CKD, and psoriasis, with the corresponding proportion attributable due to interaction ranging from 16.35% to 49.65% (Figure 1).

### 3.3 Association of MAA‐BMI phenotypes with risk of obesity‐related multimorbidity

**FIGURE 2:**
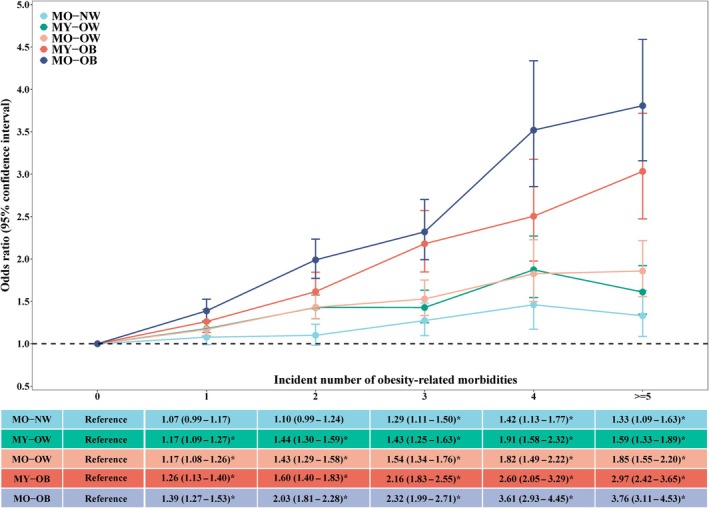
Association of metabolomic aging acceleration and body mass index phenotypes with risk of obesity‐related multimorbidity. Multinomial logistic regression was used to estimate the association of metabolomic aging acceleration and body mass index phenotypes with risk of obesity‐related multimorbidity, adjusting for age, sex, ethnicity, Townsend deprivation index, educational attainment, physical activity, healthy diet, sleep duration, smoking, drinking, and longevity genetic risk scores. Metabolomic younger normal weight was set as the reference group. The asterisk (*) indicates a significant association through two‐sided statistical tests. MO‐NW, metabolomically older normal weight; MO‐OB, metabolomically older obesity; MY‐OB, metabolomically younger obesity; MO‐OW, metabolomically older overweight; MY‐OW, metabolomically younger overweight.

Figure 2 shows the association between MAA‐BMI phenotypes and the incident number of ORMs. Compared with MY‐NW persons, participants with MO‐OB showed the highest risk of developing obesity‐related multimorbidity, followed by individuals with MY‐OB, MO‐OW, MY‐OW, and MO‐NW.

### 3.4 Effect of Metabolomic aging acceleration on risk of mortality and ORMs in different BMI categories

**FIGURE 3:**
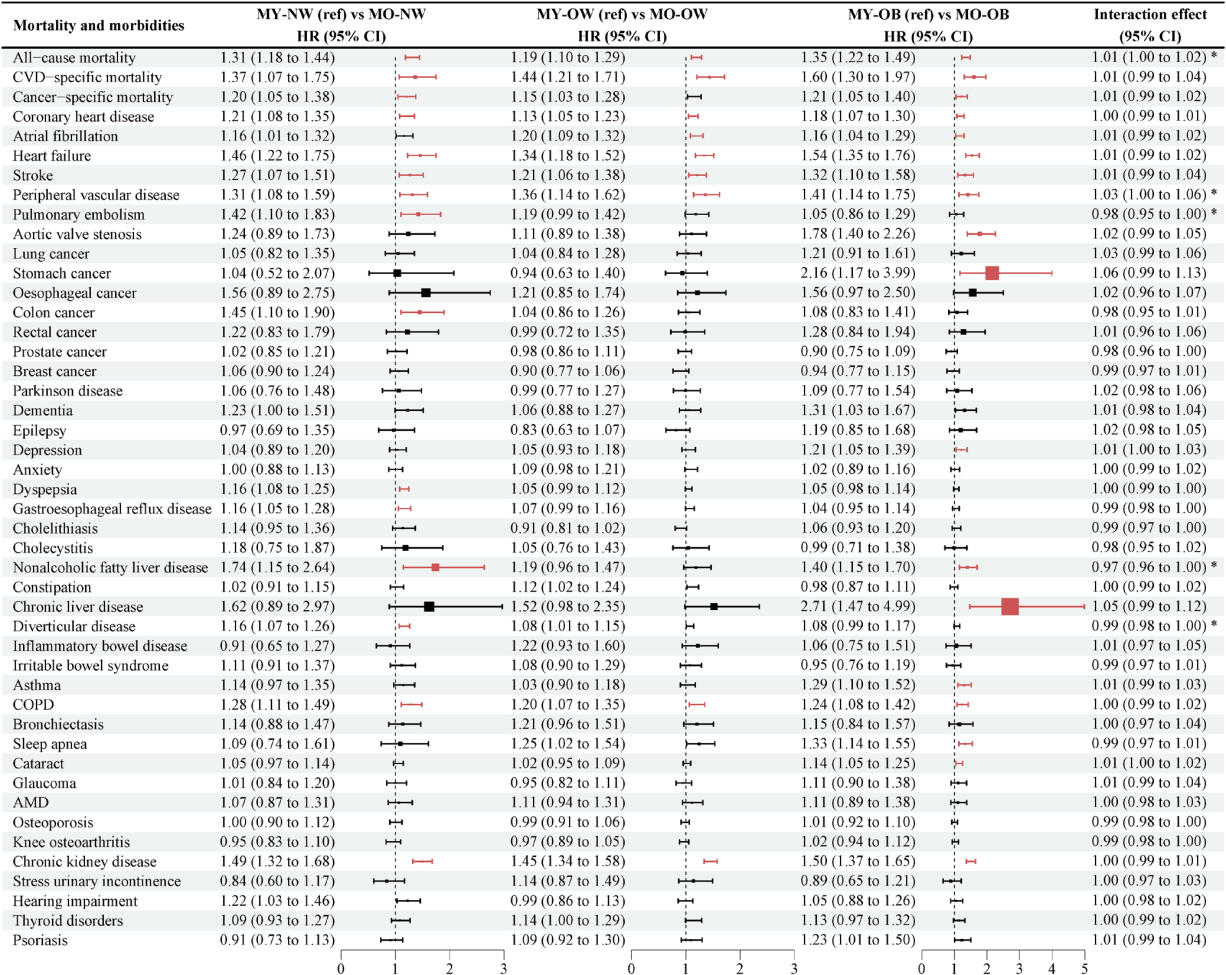
The effect of metabolomic aging acceleration on risk of mortality and obesity‐related morbidities in different BMI categories. The cox proportional hazard regression was used to estimate the effect of metabolomic aging acceleration on risk of mortality and obesity‐related morbidities in different BMI categories, adjusting for age, sex, ethnicity, Townsend deprivation index, educational attainment, physical activity, healthy diet, sleep duration, smoking, drinking, and longevity genetic risk scores. Multiplicative interaction was evaluated using HRs for the product term between the metabolomic aging status (younger or older) and body mass index (continuous). Prostate cancer was analyzed only in males. Breast cancer was analyzed only in females. Squares indicate the HRs, with black color denoting non‐significant association, red color indicating positive association, and blue color indicating inverse association. AMD, age‐related macular degeneration; CI, confidence interval; COPD, chronic obstructive pulmonary disease; CVD, cardiovascular disease; HR, hazard ratio; MO‐NW, metabolomically older normal weight; MO‐OB, metabolomically older obesity; MO‐OW, metabolomically older overweight; MY‐NW, metabolomically younger normal weight; MY‐OB, metabolomically younger obesity; MY‐OW, metabolomically younger overweight.

Figure 3 demonstrated that within each BMI category (normal weight, overweight, or obesity), individuals with metabolomic aging acceleration showed a higher risk of mortality and several ORMs after adjusting for covariates in model 2 and controlling for FDR, particularly for most CVD, chronic obstructive pulmonary disease (COPD), and CKD. Moreover, the association between metabolomic aging acceleration and all‐cause mortality (HR: 1.01; 95% CI: 1.00–1.02) and peripheral vascular disease (HR: 1.03; 95% CI: 1.00–1.06) was stronger with increasing BMI, whereas the association with pulmonary embolism (HR: 0.98; 95% CI: 0.95–1.00), NAFLD (HR: 0.97; 95% CI: 0.96–1.00), and diverticular disease (HR: 0.99; 95% CI: 0.98–1.00) was stronger with decreasing BMI.

### 3.5 The mediating role of the INFLA‐score in the association between MY‐OW/MY‐OB phenotypes and risk of mortality and obesity‐related morbidities

Figure S3 shows that both participants with MY‐OW (*β*: 1.69; 95% CI: 1.56–1.81) and MY‐OB (*β*: 3.27; 95% CI: 3.11–3.42) had significantly higher INFLA‐score compared with MY‐NW persons. Figure S4 indicates that increased INFLA‐score was associated with increased risk of mortality and various ORMs. Table 2 demonstrates that INFLA‐score partially mediated the association between the MY‐OW phenotype and the increased risk of CHD, depression, dyspepsia, gastroesophageal reflux disease (GERD), cholelithiasis, diverticular disease, and CKD, with mediation proportions of 21.90%, 23.36%, 12.03%, 11.21%, 7.94%, 16.10%, and 19.84%, respectively. Consistently, the INFLA‐score also mediated the association between the MY‐OB phenotype and the increased risk of all‐cause mortality, CVD‐specific mortality, and 10 ORMs, with mediation proportions ranging from 8.12% for cholelithiasis to 76.55% for all‐cause mortality.

**TABLE 2:** The mediating role of the INFLA‐score in the association between metabolomically younger overweight/obesity phenotypes and risk of mortality and obesity‐related morbidities.

Mortality and morbidities	Total effect	Direct effect	Mediation effect	Proportion mediated
Metabolomically younger overweight				
Coronary heart disease	0.28 (0.15 to 0.41)*	0.26 (0.13 to 0.38)*	0.06 (0.04 to 0.09)*	21.90 (8.60 to 35.19)*
Atrial fibrillation	0.13 (−0.01 to 0.27)	0.15 (0.01 to 0.29)*	−0.01 (−0.04 to 0.01)	−10.11 (−33.33 to 13.11)
Heart failure	0.24 (0.03 to 0.46)*	0.25 (0.03 to 0.46)*	0.07 (0.03 to 0.11)*	27.72 (−1.47 to 56.92)
Pulmonary embolism	0.41 (0.06 to 0.76)*	0.38 (0.04 to 0.73)*	−0.01 (−0.07 to 0.04)	−3.26 (−16.77 to 10.25)
Aortic valve stenosis	0.38 (−0.04 to 0.79)	0.34 (−0.07 to 0.75)	0.02 (−0.05 to 0.09)	6.20 (−13.47 to 25.87)
Stomach cancer	0.81 (−0.23 to 1.85)	0.81 (−0.22 to 1.84)	0.13 (−0.02 to 0.28)	16.05 (−11.65 to 43.74)
Colon cancer	0.73 (0.28 to 1.17)*	0.74 (0.30 to 1.19)*	0.07 (0.00 to 0.13)*	9.12 (−1.03 to 19.28)
Depression	0.22 (0.05 to 0.39)*	0.21 (0.03 to 0.38)*	0.05 (0.02 to 0.08)*	23.36 (0.55 to 46.17)*
Dyspepsia	0.16 (0.08 to 0.24)*	0.14 (0.06 to 0.22)*	0.02 (0.00 to 0.03)*	12.03 (0.62 to 23.44)*
Gastroesophageal reflux disease	0.30 (0.18 to 0.42)*	0.28 (0.16 to 0.40)*	0.03 (0.01 to 0.05)*	11.21 (2.91 to 19.51)*
Cholelithiasis	1.04 (0.71 to 1.37)*	1.01 (0.68 to 1.33)*	0.08 (0.04 to 0.12)*	7.94 (3.39 to 12.48)*
Cholecystitis	0.66 (−0.03 to 1.35)	0.45 (−0.19 to 1.08)	0.03 (−0.06 to 0.13)	5.06 (−10.43 to 20.54)
Nonalcoholic fatty liver disease	1.87 (0.82 to 2.92)*	1.75 (0.73 to 2.77)*	0.06 (−0.03 to 0.16)	3.45 (−1.92 to 8.82)
Diverticular disease	0.31 (0.20 to 0.41)*	0.28 (0.18 to 0.38)*	0.05 (0.03 to 0.07)*	16.10 (7.99 to 24.21)*
Asthma	0.22 (0.02 to 0.41)*	0.19 (0.00 to 0.38)	0.04 (0.01 to 0.08)*	20.27 (−4.01 to 44.55)
Sleep apnea	1.02 (0.38 to 1.67)*	1.00 (0.36 to 1.64)*	0.03 (−0.05 to 0.11)	2.73 (−5.09 to 10.56)
Cataract	0.15 (0.06 to 0.24)*	0.14 (0.05 to 0.23)*	0.01 (−0.01 to 0.03)	6.58 (−5.47 to 18.64)
Osteoporosis	0.58 (0.43 to 0.73)*	0.57 (0.42 to 0.73)*	0.02 (−0.01 to 0.04)	2.75 (−1.18 to 6.69)
Knee osteoarthritis	0.80 (0.59 to 1.01)*	0.80 (0.58 to 1.01)*	0.02 (−0.01 to 0.04)	2.18 (−1.28 to 5.65)
Chronic kidney disease	0.30 (0.15 to 0.45)*	0.26 (0.11 to 0.41)*	0.06 (0.03 to 0.09)*	19.84 (6.51 to 33.17)*
Thyroid disorders	0.17 (−0.01 to 0.35)	0.15 (−0.03 to 0.33)	0.03 (0.00 to 0.07)*	20.35 (−9.26 to 49.95)
Metabolomically younger obesity				
All‐cause mortality	0.21 (0.07 to 0.36)*	0.14 (−0.01 to 0.29)	0.16 (0.11 to 0.21)*	76.55 (20.78 to 132.33)*
CVD‐specific mortality	0.62 (0.18 to 1.06)*	0.50 (0.06 to 0.94)*	0.23 (0.10 to 0.37)*	37.53 (5.37 to 69.69)*
Cancer‐specific mortality	0.19 (0.00 to 0.39)	0.15 (−0.06 to 0.35)	0.14 (0.07 to 0.20)*	69.90 (−7.43 to 147.23)
Coronary heart disease	0.80 (0.58 to 1.01)*	0.75 (0.53 to 0.96)*	0.13 (0.08 to 0.19)*	16.53 (8.55 to 24.51)*
Atrial fibrillation	0.92 (0.65 to 1.18)*	0.95 (0.67 to 1.23)*	−0.02 (−0.08 to 0.03)	−2.58 (−8.76 to 3.60)
Heart failure	1.16 (0.75 to 1.57)*	1.05 (0.64 to 1.46)*	0.15 (0.06 to 0.25)*	13.10 (4.11 to 22.09)*
Pulmonary embolism	1.56 (0.86 to 2.25)*	1.61 (0.87 to 2.35)*	−0.01 (−0.12 to 0.11)	−0.35 (−7.74 to 7.05)
Venous thromboembolism	0.95 (0.28 to 1.62)*	0.84 (0.16 to 1.51)*	0.11 (−0.04 to 0.26)	11.51 (−6.55 to 29.58)
Aortic valve stenosis	1.09 (0.38 to 1.80)*	0.87 (0.19 to 1.55)*	0.06 (−0.10 to 0.21)	5.10 (−9.14 to 19.33)
Colon cancer	0.53 (0.04 to 1.02)*	0.65 (0.12 to 1.19)*	0.15 (0.01 to 0.29)*	28.78 (−8.60 to 66.16)
Depression	0.62 (0.35 to 0.89)*	0.54 (0.26 to 0.82)*	0.12 (0.05 to 0.19)*	19.35 (5.83 to 32.87)*
Anxiety	0.40 (0.19 to 0.60)*	0.37 (0.15 to 0.59)*	0.06 (0.00 to 0.11)*	15.00 (−0.88 to 30.88)
Dyspepsia	0.31 (0.19 to 0.42)*	0.30 (0.18 to 0.42)*	0.04 (0.01 to 0.07)*	12.70 (1.38 to 24.02)*
Gastroesophageal reflux disease	0.56 (0.38 to 0.74)*	0.53 (0.34 to 0.71)*	0.07 (0.02 to 0.11)*	12.30 (3.56 to 21.04)*
Cholelithiasis	2.27 (1.69 to 2.85)*	1.95 (1.38 to 2.51)*	0.18 (0.09 to 0.27)*	8.12 (3.68 to 12.56)*
Cholecystitis	1.72 (0.47 to 2.97)*	1.48 (0.24 to 2.72)*	0.07 (−0.14 to 0.27)	3.91 (−8.36 to 16.17)
Nonalcoholic fatty liver disease	5.13 (2.84 to 7.42)*	4.49 (2.33 to 6.65)*	0.14 (−0.07 to 0.35)	2.76 (−1.48 to 6.99)
Constipation	0.16 (0.00 to 0.32)*	0.13 (−0.04 to 0.29)	0.03 (−0.02 to 0.08)	16.87 (−17.60 to 51.35)
Diverticular disease	0.57 (0.42 to 0.73)*	0.53 (0.38 to 0.69)*	0.11 (0.07 to 0.15)*	18.86 (10.33 to 27.38)*
Asthma	0.52 (0.23 to 0.82)*	0.42 (0.12 to 0.72)*	0.11 (0.03 to 0.18)*	20.33 (1.81 to 38.85)*
COPD	0.33 (0.10 to 0.56)*	0.11 (−0.11 to 0.33)	0.25 (0.17 to 0.33)*	74.86 (21.52 to 128.20)*
Sleep apnea	5.94 (3.78 to 8.10)*	4.83 (2.94 to 6.73)*	0.04 (−0.12 to 0.20)	0.70 (−2.05 to 3.45)
Cataract	0.17 (0.05 to 0.29)*	0.14 (0.02 to 0.27)*	0.02 (−0.02 to 0.06)	11.33 (−11.30 to 33.97)
Osteoporosis	1.66 (1.36 to 1.95)*	1.67 (1.37 to 1.98)*	0.03 (−0.02 to 0.08)	1.76 (−1.08 to 4.60)
Knee osteoarthritis	2.34 (1.92 to 2.77)*	2.33 (1.89 to 2.77)*	0.04 (−0.02 to 0.09)	1.56 (−0.93 to 4.05)
Chronic kidney disease	1.05 (0.79 to 1.32)*	0.96 (0.70 to 1.23)*	0.14 (0.08 to 0.20)*	13.07 (6.52 to 19.62)*
Stress urinary incontinence	1.09 (0.35 to 1.83)*	1.26 (0.42 to 2.10)*	0.01 (−0.12 to 0.15)	1.35 (−11.08 to 13.78)
Hearing impairment	0.30 (0.04 to 0.56)*	0.31 (0.03 to 0.59)*	0.04 (−0.03 to 0.12)	14.68 (−14.01 to 43.38)
Thyroid disorders	0.58 (0.29 to 0.87)*	0.47 (0.18 to 0.77)*	0.08 (0.01 to 0.15)*	13.56 (−0.39 to 27.51)
Psoriasis	0.36 (0.04 to 0.68)*	0.26 (−0.06 to 0.59)	0.07 (−0.03 to 0.16)	18.54 (−11.97 to 49.05)

*Note*: Total effect indicated the effect of the metabolomically younger overweight/obesity phenotypes (MY‐O phenotypes) on the outcomes. Direct effect indicated the effect of the MY‐O phenotypes on the outcomes. Mediation effect indicated the effect of the MY‐O phenotypes on the outcomes acting through the INFLA‐score. Metabolomic younger normal weight was set as the reference group. Age, sex, ethnicity, Townsend deprivation index, educational attainment, physical activity, healthy diet, sleep duration, smoking, drinking, and longevity genetic risk scores are the variables included in the models for the mediators and for the outcomes. The asterisk (*) indicates a significant association by two‐tailed statistical tests.

Abbreviations: COPD, chronic obstructive pulmonary disease; CVD, cardiovascular disease.

### 3.6 Sensitivity analysis

The association of MAA‐BMI phenotypes with risk of mortality and ORMs remained largely consistent across different sex and age subgroups (Tables S2 and S3), with only a few conditions showing interaction effects. For example, males showed a stronger association with all‐cause mortality, CVD‐specific mortality, cancer‐specific mortality, PVD, and rectal cancer compared with females, while females showed a stronger association with several digestive diseases (dyspepsia, GERD, cholelithiasis, irritable bowel syndrome, chronic liver disease), depression, asthma, knee osteoarthritis, and hearing impairment compared with males. Regarding the age group, the MO‐OB phenotype showed stronger association with all‐cause mortality, CHD, AF, dementia, depression, anxiety, dyspepsia, GERD, diverticular disease, irritable bowel syndrome, COPD, osteoporosis, CKD, and psoriasis in individuals under 60 years compared with those aged 60 years and older (all *p* for interaction < 0.05). Furthermore, both sensitivity analyses—excluding individuals who developed the corresponding disease within the first 2 years of follow‐up (Figures S5–S7 and Table S4) and using a randomly selected 50% validation sample (Figure S8)—yielded results that were largely consistent with our main findings, thereby reinforcing the robustness and reliability of our conclusions.

## 4 DISCUSSION

To our knowledge, this is the first study to establish association of MAA‐BMI phenotypes with risk of mortality and a series of ORMs. This study yields three key findings. First, participants who had metabolomic aging acceleration or were outside the optimal BMI range (overweight or obesity) had worse health outcomes compared with MY‐NW group. When both adverse factors were present simultaneously (namely, the MO‐OB group), they had the highest risk of mortality and most ORMs. Second, within each BMI category (normal weight, overweight, or obesity), individuals with metabolomic aging acceleration exhibited higher mortality and cardiovascular risk. Third, chronic inflammation partially mediated the association between metabolomic younger overweight/obesity phenotypes and increased risk of adverse health outcomes.

The innovation of this study is the introduction of metabolomic aging acceleration as a novel tool to stratify mortality and ORMs risk in different BMI groups. This classification complements the existing concept of “metabolic health” and helps to stratify mortality and ORMs risk in different BMI categories. In this study, individuals with MO‐OB emerged as the most vulnerable group for mortality and most ORMs compared with MY‐NW group. Moreover, additive interactions were observed between metabolomic aging acceleration and obesity on CVD‐specific mortality and several ORMs. The dual burden of metabolomic aging acceleration and obesity likely exacerbates the deleterious effects of these health conditions, leading to a compounded risk. Additionally, the MY‐OB group also requires attention in weight management. Although this group had a lower risk of mortality and most ORMs compared to the MO‐OB group, it still faces elevated health risks compared to individuals with MY‐NW. Therefore, early weight loss interventions are necessary for individuals with obesity to reduce the risk of future health complications, even without accelerated metabolomic aging. Weight management strategies such as calorie restriction or regular exercise provide significant benefits by reducing health risks associated with excess weight and have also proven effective in improving metabolite profiles and slowing the acceleration of metabolomic aging (Laimer et al., 2016; Robinson et al., 2020; Sala et al., 2015). For example, findings from a 2‐year caloric restriction trial provided new evidence of persistent metabolic slowing accompanied by reduced oxidative stress, which further supported the rate of living and oxidative damage theories of aging in mammals (Redman et al., 2018).

On the other hand, the establishment of MAA‐BMI phenotypes sheds light on elucidating the pathophysiological basis for heterogeneity in the same BMI category. In this study, when we compared persons with the same BMI category, those with metabolomic aging acceleration exhibited higher risk of mortality and several ORMs (in particular, most CVDs, COPD, and CKD). It has been reported that the acceleration of cellular processes observed in some ORMs is related to those occurring during normal aging (Ahima, 2009). Thus, discrepancies in metabolomic aging acceleration may explain the heterogeneity of health risks in same BMI range. Furthermore, our findings may contribute to explain the “obesity paradox”, a phenomenon where some studies reported that overweight individuals have significantly lower all‐cause mortality compared to those with normal weight (Flegal et al., 2013). Our study observed that MO‐NW individuals had higher risk of mortality compared with MY‐NW persons. In contrast, MY‐OW individuals, despite having excess adiposity, did not show a significantly increased risk of mortality compared to the MY‐NW group. These findings underscore the importance of slowing accelerated metabolomic aging, even within the normal BMI range. Delaying metabolic aging acceleration can be achieved through lifestyle modifications that promote a healthier and younger metabolic profile. Regular physical activity and nutritional strategies (calorie restriction, intermittent fasting) are recognized as efficient approaches for delaying and reversing aging and age‐related diseases (Armstrong et al., 2022; Denham & Sellami, 2021; Jia et al., 2023; Lancaster & Febbraio, 2014; Takahara & Shimomura, 2014), as they are also effective in maintaining metabolic health by adjusting mitochondrial function and activating immunomodulatory mechanisms in individuals with accelerated aging and age‐related metabolic conditions (Jia et al., 2023; Lancaster & Febbraio, 2014).

Chronic inflammation offers a plausible biological mechanism explaining how excess adiposity increases health risks even without accelerated metabolomic aging. We found that individuals with overweight and obesity without accelerated metabolomic aging still exhibited low‐grade systemic inflammation. Moreover, this inflammatory state acted as a significant mediator, increasing the risk of adverse health outcomes such as all‐cause mortality, coronary heart disease, depression, and COPD. Over recent decades, there has been a significant evolution in our comprehension of the chronic low‐grade inflammation associated with obesity (Gregor & Hotamisligil, 2011; Santos & Sinha, 2021). The expansion of white adipose tissue stimulates the release of inflammation‐associated adipokines and recruits immune cells (Fuster et al., 2016), which disrupts insulin signaling and contributes to the metabolic dysfunction observed in obesity (Tanti et al., 2012). Therefore, although some individuals with overweight or obesity in this study do not exhibit metabolic age acceleration, they already show significantly elevated inflammation compared to their normal‐weight counterparts. This suggests that the “metabolomic younger” state observed in individuals with overweight and obesity may be transient, supported by previous research indicating that being “metabolically healthy” can be a temporary rather than stable phenotype in many patients with obesity (Eckel et al., 2018). Therefore, further research is warranted to explore the metabolomic aging transitions in individuals with metabolomic younger overweight and obesity and their impact on long‐term health.

In this study, we also observed several conditions with significant sex‐specific interaction effects. For instance, males with metabolomically aging acceleration or obesity showed a stronger association with all‐cause mortality, cancer‐specific mortality, peripheral vascular disease, and rectal cancer compared to females. This aligns with previous studies indicating that males with higher BMI have increased cardiovascular and cancer‐related mortality compared to females (Song et al., 2014; Yang et al., 2023). One possible mechanism is the difference in body composition and fat distribution between male and female, with higher levels of visceral adipose tissue in males contributing to an elevated risk of cardiometabolic diseases. Conversely, females showed a stronger association with several digestive diseases, depression, asthma, knee osteoarthritis, and hearing impairment compared to males. Research also suggests that obese females may be more susceptible to psychological dysfunction and some functional gastrointestinal disorders than males, possibly due to heightened societal pressure on females to maintain a thin physique (Bouchoucha et al., 2016; Carpenter et al., 2000). These observations warrant further investigation to better understand sex differences in metabolic aging and disease susceptibility.

These study has significant clinical implications. Our findings identify individuals with MO‐OB as a group that would benefit most from weight loss interventions and highlights the need for early weight interventions for this group. Besides, weight management should also be extended to individuals with overweight and obesity even in the absence of accelerated metabolomic aging, as these groups also represent increased healthy risk compared to those with MY‐NW. Finally, interventions delaying metabolomic aging acceleration are necessary for all MO groups to improve health outcome, even in normal‐weight individuals. The main strength of this study is that we are the first to establish the association between MAA‐BMI phenotypes and the risk of mortality and a range of ORMs using comprehensive data from the UK Biobank, with a large sample size and long‐term follow‐up. However, this study has several limitations. First, some covariate information, such as lifestyle factors, was self‐reported and subject to recall bias and misclassification. Second, the data on chronic conditions was linked to inpatient hospital records, potentially missing mild or undiagnosed cases. Third, due to the limited sample size for reassessing metabolomic profiles during follow‐up, we relied solely on baseline measurements and did not account for changes in metabolomic aging status over time. Fourth, despite excluding participants diagnosed with diseases within the first 2 years of follow‐up, reverse causality and residual confounding remain limitations inherent to observational studies.

## 5 CONCLUSION

In conclusion, metabolomic aging acceleration might be a novel tool to stratify mortality and ORMs risk in different BMI groups. Weight management should also be extended to individuals with overweight and obesity even in the absence of accelerated metabolomic aging, as they still face increased healthy risk compared with MY‐NW individuals. Additionally, delaying metabolic aging acceleration is needed for all metabolomically older groups, including those of normal weight.

## AUTHOR CONTRIBUTIONS

Conceptualization: XMZ, XS, HY, and MH; Data curation: XMZ, XS, HY, MH, and DS; Formal analysis: XMZ, and XS; Funding acquisition: XMZ, XS, HY, and MH; Investigation: XMZ, and XS; Methodology: XMZ, XS; Project administration: XS, HY, and MH; Supervision: XS, HY, and MH; Visualization: XMZ; Writing—original draft: XMZ, and RC; Writing—review and editing: All authors.

## FUNDING INFORMATION

The study was supported by the Global STEM Professorship Scheme (P0046113) and Henry G. Leong Endowed Professorship in Elderly Vision Health, the Start‐up Fund for RAPs under the Strategic Hiring Scheme (P0053191), National Natural Science Foundation of China (82171075, 82301260), Basic and Applied Basic Research Foundation of Guangdong Province (2023B1515120028), Brolucizumab Efficacy and Safety Single‐Arm Descriptive Trial in Patients with Persistent Diabetic Macular Edema (BEST) (2024‐29), High‐level Talent Flexible Introduction Fund of Guangdong Provincial People’s Hospital (KJ012019530), China Scholarship Council (202308440506), and Science and Technology Program of Guangzhou (20220610092).

## CONFLICT OF INTEREST STATEMENT

None declared.

## CONSENT FOR PUBLICATION

Informed consent for publication was provided from all participants.

## Supporting information


Appendix S1.

## Data Availability

Data are available in a public, open access repository (https://www.ukbiobank.ac.uk/).
