# Supplementary material for: Association of metabolomic aging acceleration and body mass index phenotypes with mortality and obesity‐related morbidities
Source: Aging Cell. 2024 Dec 12;24(4):e14435. doi: 10.1111/acel.14435 (PMC11984667; doi:10.1111/acel.14435)
Supplement: Supplementary file 1 — Appendix S1. [file ACEL-24-e14435-s001.docx]

**Association of Metabolomic Aging Acceleration and Body Mass Index Phenotypes with Mortality and Obesity-Related Morbidities**

**Supplementary Materials**

**Supplementary Figures**

sFigure 1. Flowchart of participants included in the study

sFigure 2. Associations of metabolomic aging acceleration and body mass index phenotypes with risk of mortality and obesity-related morbidities (Model 1)

sFigure 3. Associations of metabolomically younger phenotypes of overweight/obesity with INFLA-score level

sFigure 4. Association of INFLA-score with risk of mortality and obesity-related morbidities

sFigure 5. Association of metabolomic aging acceleration and body mass index phenotypes with risk of mortality and obesity-related morbidities by excluding those developed the corresponding disease within the first two years of follow-up

sFigure 6. Association of metabolomic aging acceleration and body mass index phenotypes with risk of obesity-related multimorbidity by excluding those developed the corresponding disease within the first two years of follow-up

sFigure 7. The effect of metabolomic aging acceleration on risk of mortality and obesity-related morbidities in different BMI categories by excluding those developed the corresponding disease within the first two years of follow-up

sFigure 8. Association of metabolomic aging acceleration and body mass index phenotypes with risk of mortality and obesity-related morbidities using a randomly selected 50% validation sample

**Supplementary Tables**

sTable 1. The follow-up duration and incident cases of the outcomes

sTable 2. Association of metabolomic aging acceleration and body mass index phenotypes with risk of mortality and obesity-related morbidities stratified by sex

sTable 3. Associations of metabolomic aging acceleration and body mass index phenotypes with risk of mortality and obesity-related morbidities stratified by age

sTable 4. The mediating role of the INFLA-score in the association between metabolomically younger overweight/obesity phenotypes and risk of mortality and obesity-related morbidities by excluding those developed the corresponding disease within the first two years of follow-up

sTable 5. ICD codes for diseases of interest

sTable 6. Self-reported codes for diseases of interest


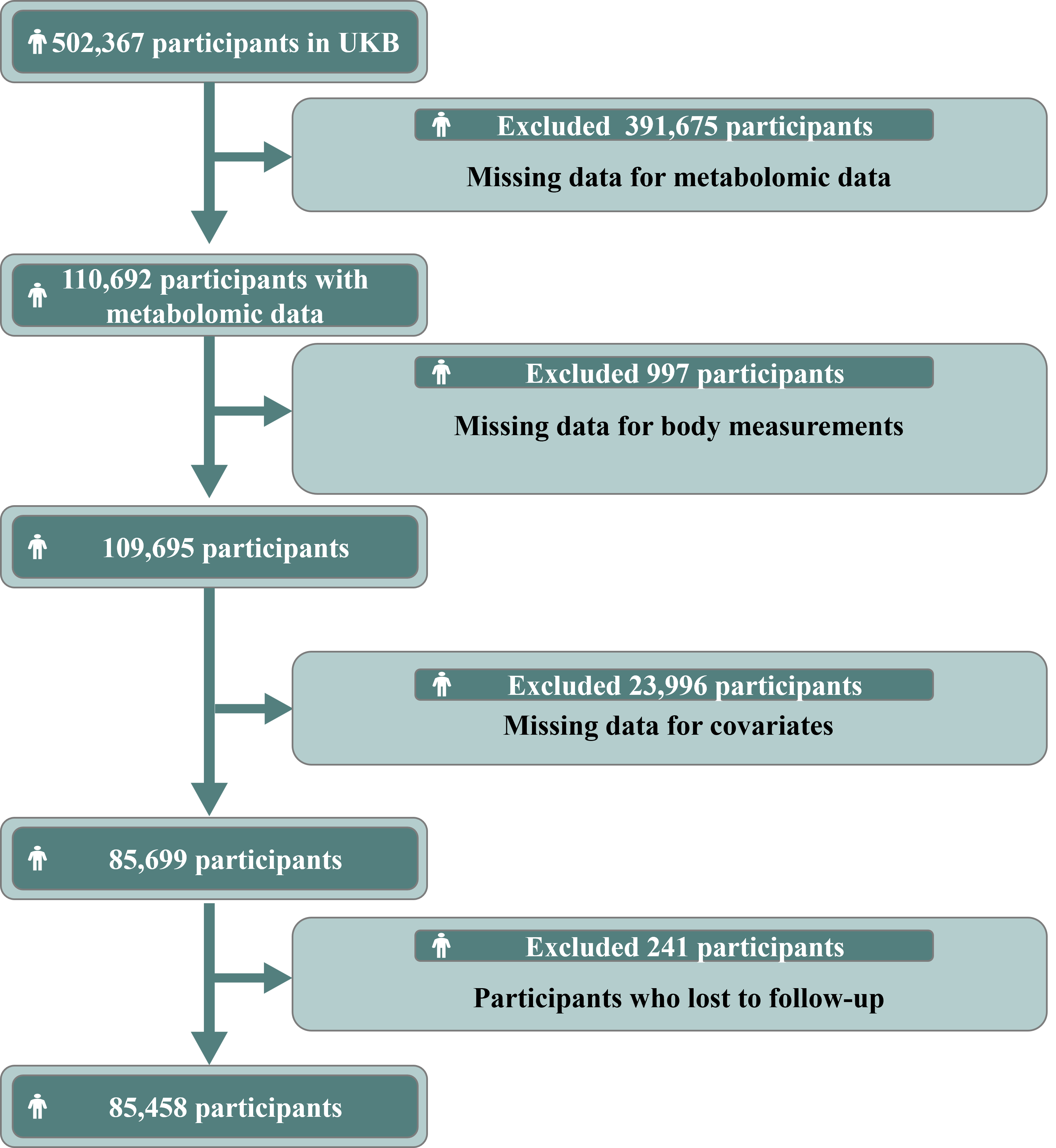


**sFigure 1. Flowchart of participants included in the study**

Abbreviations: UKB, United Kingdom biobank; TDI, Townsend deprivation index

| **sTable 1. The follow-up duration and incident cases of the outcomes** | | | | |
| --- | --- | --- | --- | --- |
| Outcomes | Total participants | Mean follow-up duration  (years) | Incident cases | Incident rate |
| All-cause mortality | 85,458 | 12.61 | 5,898 | 6.90 |
| CVD-specific mortality | 85,458 | 12.61 | 1,197 | 1.48 |
| Cancer-specific mortality | 85,458 | 12.61 | 2,959 | 3.46 |
| Coronary heart disease | 81,169 | 12.54 | 5,533 | 6.82 |
| Atrial fibrillation | 84,845 | 12.56 | 3,945 | 4.65 |
| Heart failure | 84,989 | 12.58 | 2,452 | 2.89 |
| Stroke | 84,056 | 12.59 | 1,927 | 2.29 |
| Peripheral vascular disease | 85,011 | 12.59 | 1,310 | 1.54 |
| Pulmonary embolism | 84,762 | 12.59 | 1,108 | 1.31 |
| Aortic valve stenosis | 85,351 | 12.59 | 786 | 0.92 |
| Lung cancer | 85,385 | 12.60 | 801 | 0.94 |
| Stomach cancer | 85,415 | 12.61 | 186 | 0.22 |
| Oesophageal cancer | 85,416 | 12.61 | 250 | 0.29 |
| Colon cancer | 85,116 | 12.59 | 888 | 1.04 |
| Rectal cancer | 85,322 | 12.60 | 363 | 0.43 |
| Parkinson disease | 85,314 | 12.60 | 537 | 0.63 |
| Dementia | 85,430 | 12.59 | 1,115 | 1.31 |
| Epilepsy | 84,708 | 12.6 | 516 | 0.61 |
| Depression | 80,584 | 12.58 | 2,720 | 3.38 |
| Anxiety | 83,819 | 12.57 | 3,332 | 3.98 |
| Dyspepsia | 75,664 | 12.44 | 10,174 | 13.45 |
| Gastroesophageal reflux disease | 80,855 | 12.52 | 6,185 | 7.65 |
| Cholelithiasis | 83,169 | 12.57 | 2,645 | 3.18 |
| Cholecystitis | 85,177 | 12.60 | 381 | 0.45 |
| Nonalcoholic fatty liver disease | 85,374 | 12.59 | 927 | 1.09 |
| Constipation | 84,483 | 12.57 | 3,908 | 4.63 |
| Chronic liver disease | 85,255 | 12.60 | 193 | 0.23 |
| Diverticular disease | 83,142 | 12.48 | 8,620 | 10.37 |
| Inflammatory bowel disease | 84,516 | 12.60 | 495 | 0.59 |
| Irritable bowel syndrome | 83,312 | 12.59 | 1,173 | 1.41 |
| Asthma | 75,999 | 12.58 | 2,105 | 2.77 |
| COPD | 84,090 | 12.58 | 2,699 | 3.21 |
| Bronchiectasis | 85,260 | 12.59 | 711 | 0.83 |
| Sleep apnea | 84,911 | 12.59 | 1,207 | 1.42 |
| Cataract | 83,388 | 12.50 | 7,734 | 9.27 |
| Glaucoma | 84,525 | 12.59 | 1,562 | 1.85 |
| AMD | 85,317 | 12.59 | 1,278 | 1.50 |
| Osteoporosis | 77,987 | 12.5 | 6,060 | 7.77 |
| Knee osteoarthritis | 83,871 | 12.54 | 4,835 | 5.76 |
| Chronic kidney disease | 85,012 | 12.55 | 5,419 | 6.37 |
| Stress urinary incontinence | 84,705 | 12.59 | 533 | 0.63 |
| Hearing impairment | 85,251 | 12.58 | 1,883 | 2.21 |
| Thyroid disorders | 80,854 | 12.58 | 2,223 | 2.75 |
| Psoriasis | 82,217 | 12.59 | 1,261 | 1.53 |
| Prostate cancer | 40,663 | 12.54 | 1,979 | 4.87 |
| Breast cancer | 42,477 | 12.58 | 1,634 | 3.85 |
| Individuals with corresponding diseases at baseline were excluded. Prostate cancer was analyzed only in male. Breast cancer was analyzed only in female. Abbreviations: CVD, cardiovascular disease; COPD, chronic obstructive pulmonary disease; AMD, age-related macular degeneration. | | | | |


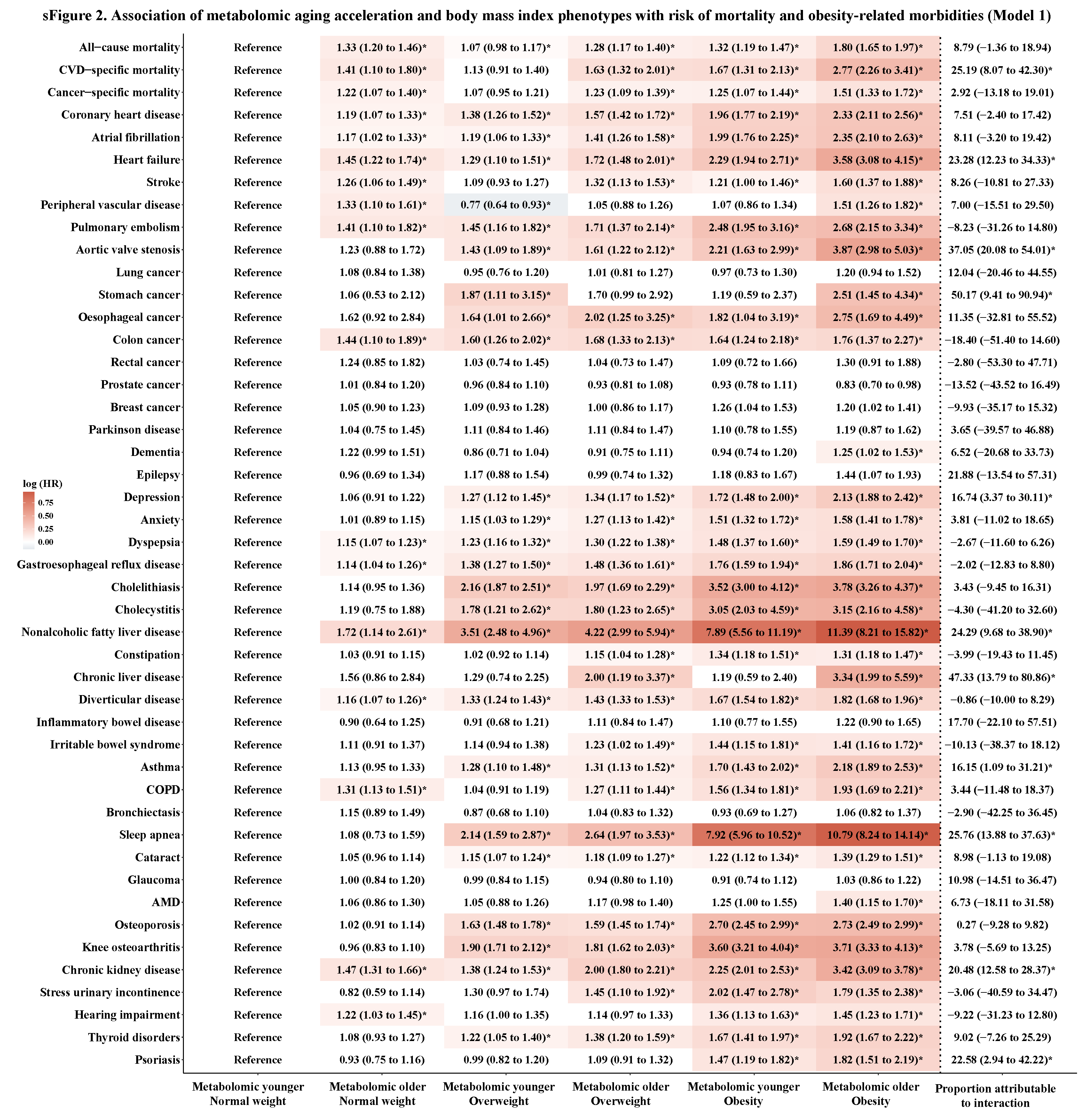


**sFigure 2. Associations of metabolomic aging acceleration and body mass index phenotypes with risk of mortality and obesity-related morbidities (Model 1)**

The cox proportional hazard regression was used to estimate the association of metabolomic aging acceleration and body mass index phenotypes with risk of mortality and obesity-related morbidities, adjusting for age and sex. Metabolomic younger normal weight was set as the reference group. Prostate cancer was analyzed only in males. Breast cancer was analyzed only in females. Proportion attributable due to interaction and corresponding 95% confidence intervals was used as the measure of additive interaction between the metabolomic aging acceleration (younger vs older) and obesity status (normal weight vs obesity), and the additive interaction was statistically significant when its confidence interval did not include 0. The asterisk (*) indicates a significant association through two-sided statistical tests.

Abbreviations: HR, hazard ratio; CVD, cardiovascular disease; COPD, chronic obstructive pulmonary disease; AMD, age-related macular degeneration.


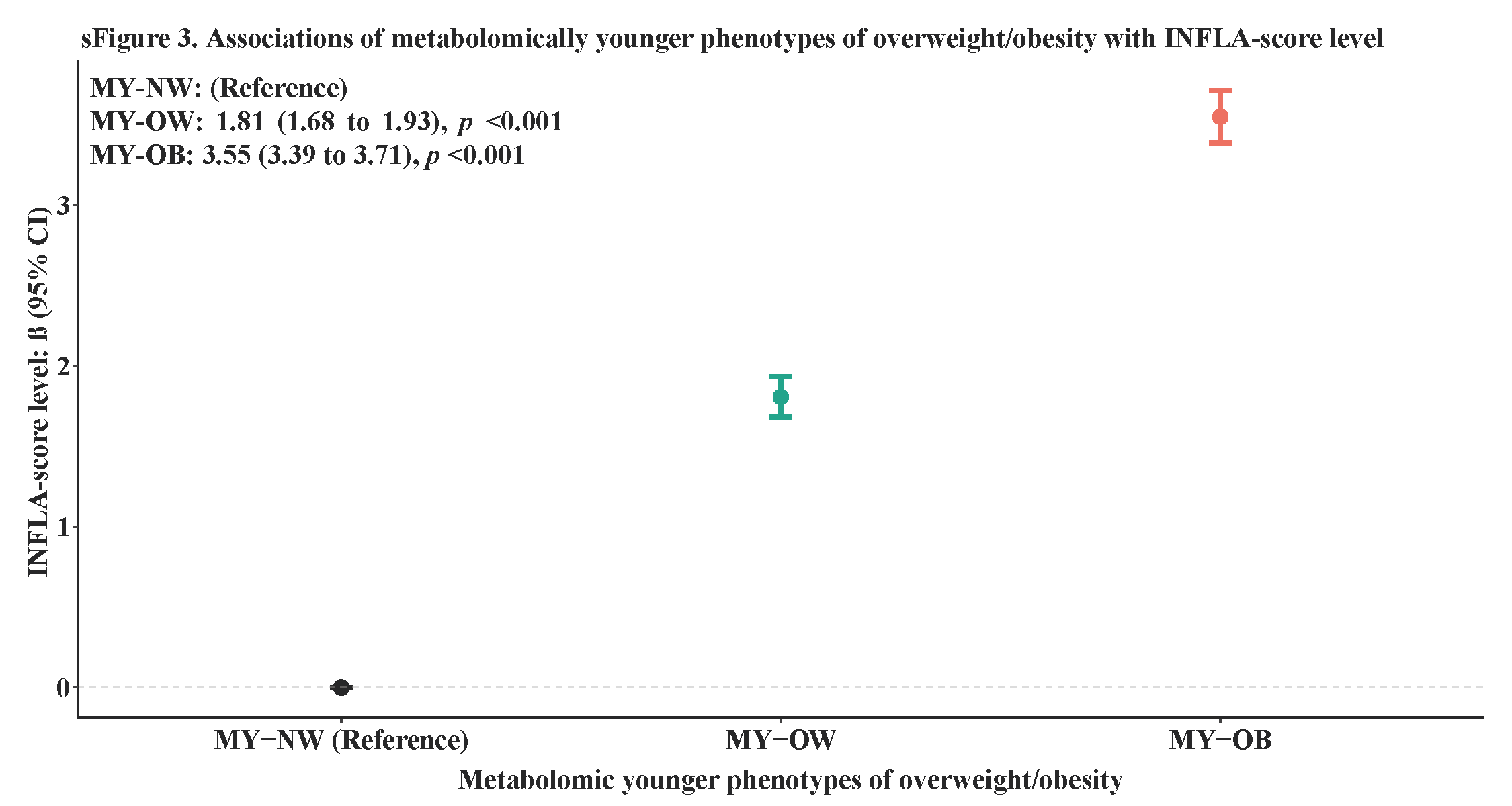


**sFigure 3. Associations of metabolomically younger phenotypes of overweight/obesity with INFLA-score level**

Multiple linear regression was used to estimate the associations of metabolomically younger phenotypes of overweight/obesity with INFLA-score level, adjusting for age, sex, ethnicity, Townsend deprivation index, educational attainment, physical activity, healthy diet, sleep duration, smoking, drinking, and longevity genetic risk scores. metabolomically younger-normal weight was set as the reference group.

Abbreviations: MY-NW, metabolomically younger-normal weight; MY-OW, metabolomically younger-overweight; MY-OB, metabolomically younger-obesity; CI, confidence interval.


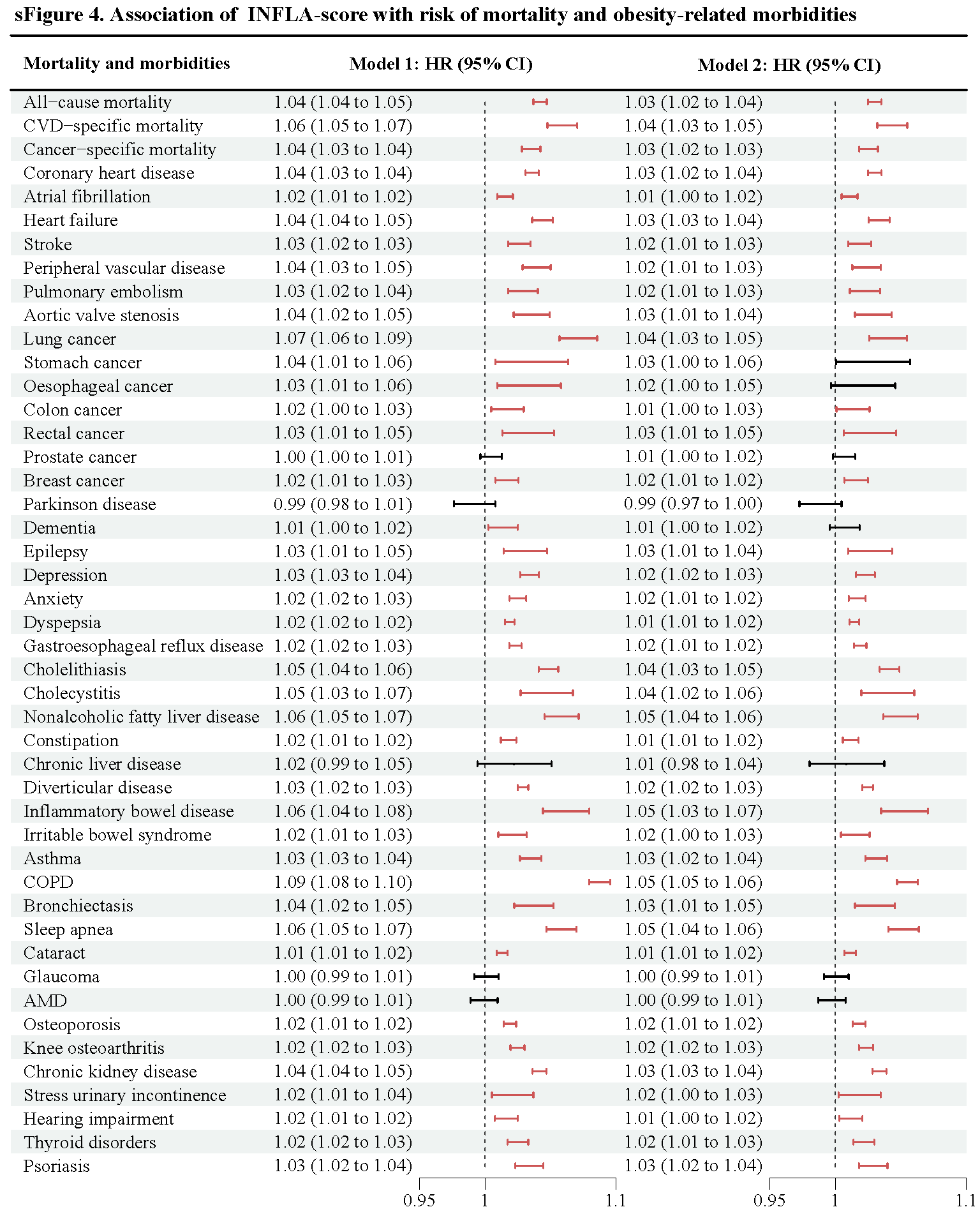


**sFigure 4. Association of INFLA-score with risk of mortality and obesity-related morbidities**

The cox proportional hazard regression was used to estimate the associations of INFLA-score with risk of mortality and obesity-related morbidities, adjusting for age and sex, ethnicity, Townsend deprivation index, educational attainment, physical activity, healthy diet, sleep duration, smoking, drinking, and longevity genetic risk scores. Prostate cancer was analyzed only in males. Breast cancer was analyzed only in females. Squares indicate the hazard ratios, with black color denoting non-significant associations, red color indicating positive associations, and blue color indicating inverse associations.

Abbreviations: HR, hazard ratio; CI, confidence interval; CVD, cardiovascular disease; COPD, chronic obstructive pulmonary disease; AMD, age-related macular degeneration.

| **sTable 2. Association of metabolomic aging acceleration and body mass index phenotypes with risk of mortality and obesity-related morbidities stratified by sex** | | | |
| --- | --- | --- | --- |
| **Mortality and obesity-related morbidities** | **Male** | **Female** | ***p* for interaction** |
|  | **HR (95%CI)** | **HR (95%CI)** |  |
| **All-cause mortality** |  |  |  |
| MY-NW | Reference | Reference | - |
| MO-NW | 1.37 (1.19 to 1.58)* | 1.25 (1.08 to 1.44)* | 0.345 |
| MY-OW | 1.10 (0.98 to 1.23) | 0.99 (0.85 to 1.15) | 0.148 |
| MO-OW | 1.37 (1.22 to 1.54)* | 1.08 (0.94 to 1.24) | **0.004** |
| MY-OB | 1.19 (1.04 to 1.36)* | 1.28 (1.07 to 1.52)* | 0.896 |
| MO-OB | 1.78 (1.58 to 2.01)* | 1.40 (1.21 to 1.61)* | **0.002** |
| **CVD-specific mortality** |  |  |  |
| MY-NW | Reference | Reference | - |
| MO-NW | 1.44 (1.05 to 1.98)* | 1.25 (0.85 to 1.83) | 0.568 |
| MY-OW | 1.23 (0.95 to 1.60) | 0.90 (0.60 to 1.35) | 0.194 |
| MO-OW | 1.81 (1.40 to 2.35)* | 1.13 (0.78 to 1.64) | **0.037** |
| MY-OB | 1.64 (1.22 to 2.20)* | 1.32 (0.83 to 2.09) | 0.404 |
| MO-OB | 2.77 (2.14 to 3.58)* | 1.86 (1.29 to 2.69)* | 0.063 |
| **Cancer-specific mortality** |  |  |  |
| MY-NW | Reference | Reference | - |
| MO-NW | 1.27 (1.04 to 1.56)* | 1.16 (0.97 to 1.40) | 0.504 |
| MY-OW | 1.07 (0.90 to 1.26) | 1.07 (0.89 to 1.30) | 0.738 |
| MO-OW | 1.32 (1.11 to 1.56)* | 1.10 (0.92 to 1.31) | 0.074 |
| MY-OB | 1.16 (0.95 to 1.42) | 1.22 (0.97 to 1.53) | 0.891 |
| MO-OB | 1.55 (1.30 to 1.85)* | 1.26 (1.04 to 1.52)* | **0.047** |
| **Coronary heart disease** |  |  |  |
| MY-NW | Reference | Reference | - |
| MO-NW | 1.21 (1.04 to 1.41)* | 1.19 (1.01 to 1.41)* | 0.885 |
| MY-OW | 1.37 (1.22 to 1.54)* | 1.25 (1.06 to 1.47)* | 0.456 |
| MO-OW | 1.45 (1.29 to 1.64)* | 1.64 (1.41 to 1.91)* | 0.185 |
| MY-OB | 1.77 (1.55 to 2.03)* | 1.92 (1.60 to 2.30)* | 0.343 |
| MO-OB | 2.13 (1.87 to 2.41)* | 2.22 (1.90 to 2.58)* | 0.613 |
| **Atrial fibrillation** |  |  |  |
| MY-NW | Reference | Reference | - |
| MO-NW | 1.17 (0.98 to 1.41) | 1.13 (0.93 to 1.37) | 0.784 |
| MY-OW | 1.19 (1.03 to 1.38)* | 1.07 (0.88 to 1.30) | 0.508 |
| MO-OW | 1.43 (1.24 to 1.66)* | 1.26 (1.05 to 1.51)* | 0.353 |
| MY-OB | 1.84 (1.57 to 2.16)* | 1.93 (1.57 to 2.38)* | 0.550 |
| MO-OB | 2.25 (1.94 to 2.61)* | 2.06 (1.73 to 2.47)* | 0.575 |
| **Heart failure** |  |  |  |
| MY-NW | Reference | Reference | - |
| MO-NW | 1.46 (1.15 to 1.86)* | 1.42 (1.08 to 1.86)* | 0.911 |
| MY-OW | 1.26 (1.04 to 1.54)* | 1.18 (0.90 to 1.56) | 0.741 |
| MO-OW | 1.69 (1.39 to 2.05)* | 1.56 (1.22 to 2.01)* | 0.686 |
| MY-OB | 2.02 (1.64 to 2.50)* | 2.09 (1.56 to 2.79)* | 0.898 |
| MO-OB | 3.18 (2.63 to 3.85)* | 3.11 (2.44 to 3.96)* | 0.813 |
| **Stroke** |  |  |  |
| MY-NW | Reference | Reference | - |
| MO-NW | 1.28 (1.01 to 1.61)* | 1.27 (0.98 to 1.65) | 0.995 |
| MY-OW | 0.97 (0.80 to 1.18) | 1.28 (0.99 to 1.65) | 0.088 |
| MO-OW | 1.19 (0.98 to 1.45) | 1.48 (1.16 to 1.88)* | 0.164 |
| MY-OB | 1.12 (0.89 to 1.42) | 1.11 (0.80 to 1.55) | 0.982 |
| MO-OB | 1.48 (1.20 to 1.82)* | 1.56 (1.21 to 2.03)* | 0.675 |
| **Peripheral vascular disease** |  |  |  |
| MY-NW | Reference | Reference | - |
| MO-NW | 1.64 (1.23 to 2.20)* | 1.08 (0.84 to 1.40) | **0.028** |
| MY-OW | 0.94 (0.72 to 1.21) | 0.63 (0.47 to 0.84)* | **0.027** |
| MO-OW | 1.36 (1.05 to 1.75)* | 0.76 (0.59 to 1.00)* | **0.001** |
| MY-OB | 1.31 (0.98 to 1.76) | 0.64 (0.44 to 0.95)* | **0.002** |
| MO-OB | 1.91 (1.47 to 2.47)* | 0.89 (0.67 to 1.18) | **<0.001** |
| **Pulmonary embolism** |  |  |  |
| MY-NW | Reference | Reference | - |
| MO-NW | 1.67 (1.15 to 2.41)* | 1.23 (0.87 to 1.74) | 0.248 |
| MY-OW | 1.57 (1.15 to 2.12)* | 1.16 (0.82 to 1.66) | 0.212 |
| MO-OW | 1.62 (1.18 to 2.22)* | 1.75 (1.28 to 2.40)* | 0.746 |
| MY-OB | 1.95 (1.38 to 2.75)* | 3.13 (2.23 to 4.41)* | 0.077 |
| MO-OB | 2.20 (1.59 to 3.03)* | 3.01 (2.21 to 4.10)* | 0.266 |
| **Aortic valve stenosis** |  |  |  |
| MY-NW | Reference | Reference | - |
| MO-NW | 1.05 (0.67 to 1.67) | 1.45 (0.89 to 2.38) | 0.373 |
| MY-OW | 1.34 (0.95 to 1.89) | 1.50 (0.93 to 2.43) | 0.656 |
| MO-OW | 1.45 (1.02 to 2.06)* | 1.78 (1.13 to 2.81)* | 0.472 |
| MY-OB | 2.12 (1.46 to 3.08)* | 1.84 (1.06 to 3.18)* | 0.829 |
| MO-OB | 3.62 (2.59 to 5.05)* | 3.49 (2.26 to 5.41)* | 0.976 |
| **Lung cancer** |  |  |  |
| MY-NW | Reference | Reference | - |
| MO-NW | 1.05 (0.72 to 1.53) | 1.03 (0.74 to 1.45) | 0.919 |
| MY-OW | 0.86 (0.63 to 1.16) | 0.97 (0.69 to 1.37) | 0.621 |
| MO-OW | 0.89 (0.65 to 1.22) | 1.03 (0.74 to 1.42) | 0.571 |
| MY-OB | 0.89 (0.61 to 1.28) | 0.72 (0.45 to 1.16) | 0.464 |
| MO-OB | 0.85 (0.60 to 1.20) | 1.21 (0.86 to 1.71) | 0.152 |
| **Stomach cancer** |  |  |  |
| MY-NW | Reference | Reference | - |
| MO-NW | 0.87 (0.35 to 2.19) | 1.37 (0.46 to 4.09) | 0.556 |
| MY-OW | 2.00 (1.08 to 3.68)* | 1.02 (0.33 to 3.19) | 0.339 |
| MO-OW | 1.61 (0.84 to 3.08) | 1.80 (0.67 to 4.82) | 0.819 |
| MY-OB | 1.00 (0.43 to 2.28) | 1.32 (0.37 to 4.72) | 0.576 |
| MO-OB | 2.59 (1.35 to 4.97)* | 1.53 (0.52 to 4.48) | 0.533 |
| **Oesophageal cancer** |  |  |  |
| MY-NW | Reference | Reference | - |
| MO-NW | 1.06 (0.47 to 2.35) | 2.30 (0.98 to 5.37) | 0.210 |
| MY-OW | 1.72 (0.97 to 3.06) | 1.06 (0.40 to 2.84) | 0.388 |
| MO-OW | 1.84 (1.02 to 3.31)* | 2.15 (0.94 to 4.93) | 0.816 |
| MY-OB | 1.93 (1.01 to 3.69)* | 0.59 (0.13 to 2.81) | 0.152 |
| MO-OB | 2.83 (1.57 to 5.11)* | 1.55 (0.59 to 4.05) | 0.236 |
| **Colon cancer** |  |  |  |
| MY-NW | Reference | Reference | - |
| MO-NW | 1.15 (0.75 to 1.76) | 1.66 (1.16 to 2.37)* | 0.192 |
| MY-OW | 1.52 (1.11 to 2.09)* | 1.64 (1.15 to 2.35)* | 0.839 |
| MO-OW | 1.63 (1.18 to 2.27)* | 1.69 (1.19 to 2.38)* | 0.971 |
| MY-OB | 1.67 (1.15 to 2.42)* | 1.36 (0.85 to 2.16) | 0.434 |
| MO-OB | 1.82 (1.29 to 2.58)* | 1.50 (1.02 to 2.20)* | 0.397 |
| **Rectal cancer** |  |  |  |
| MY-NW | Reference | Reference | - |
| MO-NW | 1.52 (0.89 to 2.60) | 1.01 (0.58 to 1.74) | 0.279 |
| MY-OW | 1.15 (0.73 to 1.79) | 0.94 (0.54 to 1.63) | 0.585 |
| MO-OW | 1.17 (0.73 to 1.86) | 0.91 (0.54 to 1.55) | 0.516 |
| MY-OB | 1.38 (0.82 to 2.32) | 0.50 (0.20 to 1.21) | 0.059 |
| MO-OB | 1.91 (1.20 to 3.05)* | 0.40 (0.19 to 0.86)* | **0.001** |
| **Parkinson disease** |  |  |  |
| MY-NW | Reference | Reference | - |
| MO-NW | 1.38 (0.91 to 2.10) | 0.73 (0.43 to 1.26) | 0.066 |
| MY-OW | 1.16 (0.83 to 1.64) | 1.00 (0.62 to 1.62) | 0.614 |
| MO-OW | 1.08 (0.75 to 1.56) | 1.17 (0.75 to 1.83) | 0.818 |
| MY-OB | 0.98 (0.63 to 1.53) | 1.26 (0.72 to 2.23) | 0.475 |
| MO-OB | 1.22 (0.82 to 1.81) | 1.00 (0.60 to 1.67) | 0.578 |
| **Dementia** |  |  |  |
| MY-NW | Reference | Reference | - |
| MO-NW | 1.31 (0.97 to 1.76) | 1.16 (0.87 to 1.56) | 0.618 |
| MY-OW | 0.85 (0.66 to 1.08) | 0.79 (0.58 to 1.07) | 0.743 |
| MO-OW | 0.86 (0.66 to 1.12) | 0.89 (0.67 to 1.19) | 0.777 |
| MY-OB | 0.77 (0.56 to 1.06) | 1.00 (0.69 to 1.45) | 0.309 |
| MO-OB | 1.23 (0.94 to 1.62) | 0.93 (0.68 to 1.28) | 0.188 |
| **Epilepsy** |  |  |  |
| MY-NW | Reference | Reference | - |
| MO-NW | 0.94 (0.59 to 1.52) | 0.99 (0.62 to 1.58) | 0.887 |
| MY-OW | 1.01 (0.70 to 1.45) | 1.31 (0.85 to 2.02) | 0.290 |
| MO-OW | 0.84 (0.57 to 1.25) | 1.09 (0.71 to 1.69) | 0.345 |
| MY-OB | 0.85 (0.53 to 1.38) | 1.50 (0.89 to 2.50) | 0.091 |
| MO-OB | 1.10 (0.73 to 1.66) | 1.59 (1.03 to 2.45)* | 0.192 |
| **Depression** |  |  |  |
| MY-NW | Reference | Reference | - |
| MO-NW | 1.19 (0.91 to 1.55) | 1.01 (0.84 to 1.20) | 0.284 |
| MY-OW | 1.21 (0.98 to 1.49) | 1.25 (1.05 to 1.48)* | 0.939 |
| MO-OW | 1.32 (1.07 to 1.64)* | 1.26 (1.07 to 1.48)* | 0.569 |
| MY-OB | 1.25 (0.97 to 1.61) | 1.81 (1.50 to 2.18)* | **0.035** |
| MO-OB | 1.74 (1.39 to 2.18)* | 2.03 (1.73 to 2.38)* | 0.438 |
| **Anxiety** |  |  |  |
| MY-NW | Reference | Reference | - |
| MO-NW | 1.00 (0.78 to 1.29) | 1.01 (0.87 to 1.17) | 0.926 |
| MY-OW | 1.14 (0.94 to 1.38) | 1.11 (0.96 to 1.28) | 0.908 |
| MO-OW | 1.18 (0.97 to 1.44) | 1.25 (1.09 to 1.43)* | 0.586 |
| MY-OB | 1.31 (1.05 to 1.65)* | 1.46 (1.23 to 1.72)* | 0.473 |
| MO-OB | 1.46 (1.19 to 1.80)* | 1.44 (1.24 to 1.66)* | 0.801 |
| **Dyspepsia** |  |  |  |
| MY-NW | Reference | Reference | - |
| MO-NW | 1.23 (1.09 to 1.38)* | 1.13 (1.03 to 1.24)* | 0.275 |
| MY-OW | 1.10 (1.00 to 1.21)* | 1.28 (1.17 to 1.40)* | **0.047** |
| MO-OW | 1.20 (1.08 to 1.32)* | 1.31 (1.20 to 1.42)* | 0.217 |
| MY-OB | 1.19 (1.06 to 1.33)* | 1.55 (1.39 to 1.72)* | **0.002** |
| MO-OB | 1.27 (1.15 to 1.42)* | 1.64 (1.50 to 1.80)* | **<0.001** |
| **Gastroesophageal reflux disease** |  |  |  |
| MY-NW | Reference | Reference | - |
| MO-NW | 1.15 (0.99 to 1.35) | 1.17 (1.03 to 1.32)* | 0.875 |
| MY-OW | 1.13 (1.00 to 1.28) | 1.51 (1.34 to 1.70)* | **0.001** |
| MO-OW | 1.26 (1.11 to 1.43)* | 1.55 (1.38 to 1.74)* | **0.019** |
| MY-OB | 1.31 (1.13 to 1.52)* | 1.91 (1.67 to 2.20)* | **<0.001** |
| MO-OB | 1.31 (1.14 to 1.51)* | 2.09 (1.86 to 2.35)* | **<0.001** |
| **Cholelithiasis** |  |  |  |
| MY-NW | Reference | Reference | - |
| MO-NW | 1.09 (0.80 to 1.49) | 1.23 (0.98 to 1.54) | 0.533 |
| MY-OW | 1.55 (1.23 to 1.95)* | 2.62 (2.16 to 3.18)* | **0.001** |
| MO-OW | 1.43 (1.12 to 1.82)* | 2.37 (1.95 to 2.87)* | **0.002** |
| MY-OB | 2.57 (2.00 to 3.29)* | 3.89 (3.15 to 4.79)* | **0.013** |
| MO-OB | 2.93 (2.32 to 3.71)* | 4.01 (3.32 to 4.84)* | **0.032** |
| **Cholecystitis** |  |  |  |
| MY-NW | Reference | Reference | - |
| MO-NW | 1.29 (0.60 to 2.76) | 1.19 (0.67 to 2.11) | 0.870 |
| MY-OW | 1.50 (0.83 to 2.70) | 2.06 (1.23 to 3.43)* | 0.507 |
| MO-OW | 2.13 (1.19 to 3.81)* | 1.46 (0.85 to 2.49) | 0.297 |
| MY-OB | 2.36 (1.25 to 4.46)* | 3.45 (2.00 to 5.93)* | 0.446 |
| MO-OB | 2.44 (1.33 to 4.49)* | 3.45 (2.12 to 5.60)* | 0.427 |
| **Nonalcoholic fatty liver disease** |  |  |  |
| MY-NW | Reference | Reference | - |
| MO-NW | 1.34 (0.70 to 2.56) | 2.08 (1.20 to 3.62)* | 0.299 |
| MY-OW | 2.76 (1.70 to 4.46)* | 4.07 (2.47 to 6.71)* | 0.264 |
| MO-OW | 3.28 (2.03 to 5.32)* | 4.81 (2.96 to 7.82)* | 0.254 |
| MY-OB | 5.75 (3.52 to 9.39)* | 8.37 (5.06 to 13.84)* | 0.285 |
| MO-OB | 8.29 (5.2 to 13.23)* | 11.56 (7.25 to 18.43)* | 0.286 |
| **Constipation** |  |  |  |
| MY-NW | Reference | Reference | - |
| MO-NW | 1.06 (0.87 to 1.28) | 1.02 (0.88 to 1.18) | 0.749 |
| MY-OW | 1.02 (0.88 to 1.18) | 1.00 (0.86 to 1.15) | 0.534 |
| MO-OW | 1.12 (0.96 to 1.31) | 1.16 (1.01 to 1.33)* | 0.986 |
| MY-OB | 1.26 (1.05 to 1.50)* | 1.20 (1.01 to 1.44)* | 0.488 |
| MO-OB | 1.30 (1.11 to 1.54)* | 1.12 (0.96 to 1.30) | 0.099 |
| **Chronic liver disease** |  |  |  |
| MY-NW | Reference | Reference | - |
| MO-NW | 1.59 (0.71 to 3.55) | 1.60 (0.64 to 3.99) | 0.991 |
| MY-OW | 0.92 (0.45 to 1.89) | 2.02 (0.85 to 4.79) | 0.151 |
| MO-OW | 1.78 (0.91 to 3.47) | 2.12 (0.92 to 4.89) | 0.720 |
| MY-OB | 0.34 (0.10 to 1.23) | 2.75 (1.08 to 7.03)* | **0.007** |
| MO-OB | 3.06 (1.58 to 5.94)* | 2.56 (1.09 to 6.00)* | 0.823 |
| **Diverticular disease** |  |  |  |
| MY-NW | Reference | Reference | - |
| MO-NW | 1.16 (1.01 to 1.33)* | 1.16 (1.05 to 1.29)* | 0.961 |
| MY-OW | 1.27 (1.14 to 1.41)* | 1.31 (1.18 to 1.46)* | 0.609 |
| MO-OW | 1.34 (1.20 to 1.49)* | 1.45 (1.31 to 1.60)* | 0.249 |
| MY-OB | 1.53 (1.35 to 1.73)* | 1.65 (1.46 to 1.86)* | 0.361 |
| MO-OB | 1.66 (1.48 to 1.86)* | 1.79 (1.61 to 1.98)* | 0.326 |
| **Inflammatory bowel disease** |  |  |  |
| MY-NW | Reference | Reference | - |
| MO-NW | 0.81 (0.49 to 1.32) | 1.00 (0.64 to 1.58) | 0.517 |
| MY-OW | 0.80 (0.55 to 1.16) | 0.90 (0.57 to 1.44) | 0.648 |
| MO-OW | 0.94 (0.64 to 1.37) | 1.18 (0.77 to 1.80) | 0.347 |
| MY-OB | 0.82 (0.51 to 1.31) | 1.20 (0.70 to 2.03) | 0.199 |
| MO-OB | 0.88 (0.58 to 1.35) | 1.28 (0.82 to 1.98) | 0.105 |
| **Irritable bowel syndrome** |  |  |  |
| MY-NW | Reference | Reference | - |
| MO-NW | 1.06 (0.70 to 1.59) | 1.15 (0.91 to 1.46) | 0.686 |
| MY-OW | 0.84 (0.60 to 1.16) | 1.21 (0.96 to 1.54) | 0.062 |
| MO-OW | 0.92 (0.65 to 1.29) | 1.29 (1.03 to 1.61)* | 0.078 |
| MY-OB | 1.02 (0.69 to 1.52) | 1.41 (1.07 to 1.87)* | 0.159 |
| MO-OB | 0.80 (0.54 to 1.18) | 1.49 (1.18 to 1.89)* | **0.004** |
| **Asthma** |  |  |  |
| MY-NW | Reference | Reference | - |
| MO-NW | 1.08 (0.82 to 1.43) | 1.20 (0.97 to 1.47) | 0.512 |
| MY-OW | 1.00 (0.81 to 1.25) | 1.43 (1.17 to 1.75)* | **0.024** |
| MO-OW | 1.13 (0.90 to 1.41) | 1.37 (1.12 to 1.67)* | 0.202 |
| MY-OB | 1.34 (1.04 to 1.72)* | 1.71 (1.34 to 2.17)* | 0.207 |
| MO-OB | 1.52 (1.21 to 1.93)* | 2.44 (2.01 to 2.95)* | **0.003** |
| **COPD** |  |  |  |
| MY-NW | Reference | Reference | - |
| MO-NW | 1.33 (1.08 to 1.64)* | 1.25 (1.02 to 1.53)* | 0.693 |
| MY-OW | 0.94 (0.79 to 1.12) | 1.08 (0.88 to 1.33) | 0.449 |
| MO-OW | 1.19 (1.00 to 1.43)* | 1.17 (0.96 to 1.43) | 0.778 |
| MY-OB | 1.35 (1.11 to 1.64)* | 1.13 (0.87 to 1.45) | 0.153 |
| MO-OB | 1.55 (1.30 to 1.86)* | 1.63 (1.34 to 2.00)* | 0.971 |
| **Bronchiectasis** |  |  |  |
| MY-NW | Reference | Reference | - |
| MO-NW | 1.27 (0.81 to 1.97) | 1.08 (0.79 to 1.48) | 0.592 |
| MY-OW | 0.99 (0.69 to 1.42) | 0.71 (0.50 to 1.01) | 0.122 |
| MO-OW | 1.11 (0.77 to 1.61) | 0.95 (0.70 to 1.29) | 0.396 |
| MY-OB | 0.97 (0.63 to 1.50) | 0.68 (0.43 to 1.08) | 0.129 |
| MO-OB | 1.22 (0.83 to 1.81) | 0.70 (0.48 to 1.01) | 0.013 |
| **Sleep apnea** |  |  |  |
| MY-NW | Reference | Reference | - |
| MO-NW | 1.64 (0.99 to 2.70) | 0.61 (0.32 to 1.16) | 0.015 |
| MY-OW | 2.57 (1.73 to 3.82)* | 1.55 (0.95 to 2.54) | 0.096 |
| MO-OW | 3.15 (2.12 to 4.68)* | 2.05 (1.30 to 3.23)* | 0.122 |
| MY-OB | 8.87 (6.02 to 13.07)* | 5.60 (3.60 to 8.73)* | 0.126 |
| MO-OB | 11.85 (8.14 to 17.26)* | 7.85 (5.26 to 11.72)* | 0.128 |
| **Cataract** |  |  |  |
| MY-NW | Reference | Reference | - |
| MO-NW | 1.04 (0.90 to 1.21) | 1.05 (0.95 to 1.17) | 0.911 |
| MY-OW | 1.13 (1.01 to 1.27)* | 1.16 (1.05 to 1.28)* | 0.767 |
| MO-OW | 1.19 (1.06 to 1.33)* | 1.14 (1.04 to 1.26)* | 0.574 |
| MY-OB | 1.14 (1.00 to 1.31) | 1.20 (1.06 to 1.36)* | 0.811 |
| MO-OB | 1.43 (1.26 to 1.62)* | 1.27 (1.15 to 1.41)* | 0.081 |
| **Glaucoma** |  |  |  |
| MY-NW | Reference | Reference | - |
| MO-NW | 0.94 (0.70 to 1.25) | 1.05 (0.84 to 1.32) | 0.583 |
| MY-OW | 0.97 (0.78 to 1.20) | 0.98 (0.78 to 1.23) | 0.954 |
| MO-OW | 0.95 (0.75 to 1.20) | 0.90 (0.72 to 1.12) | 0.650 |
| MY-OB | 0.77 (0.57 to 1.03) | 1.01 (0.76 to 1.35) | 0.212 |
| MO-OB | 1.02 (0.79 to 1.32) | 0.94 (0.73 to 1.20) | 0.504 |
| **AMD** |  |  |  |
| MY-NW | Reference | Reference | - |
| MO-NW | 0.89 (0.62 to 1.30) | 1.14 (0.89 to 1.46) | 0.294 |
| MY-OW | 0.92 (0.70 to 1.21) | 1.14 (0.89 to 1.46) | 0.285 |
| MO-OW | 1.09 (0.82 to 1.45) | 1.19 (0.94 to 1.51) | 0.716 |
| MY-OB | 1.06 (0.76 to 1.48) | 1.33 (0.98 to 1.79) | 0.425 |
| MO-OB | 1.20 (0.88 to 1.63) | 1.47 (1.14 to 1.89)* | 0.435 |
| **Osteoporosis** |  |  |  |
| MY-NW | Reference | Reference | - |
| MO-NW | 0.92 (0.76 to 1.12) | 1.05 (0.92 to 1.21) | 0.252 |
| MY-OW | 1.56 (1.36 to 1.78)* | 1.62 (1.43 to 1.84)* | 0.703 |
| MO-OW | 1.59 (1.38 to 1.83)* | 1.53 (1.35 to 1.73)* | 0.726 |
| MY-OB | 2.49 (2.14 to 2.89)* | 2.81 (2.45 to 3.23)* | 0.247 |
| MO-OB | 2.57 (2.23 to 2.97)* | 2.79 (2.47 to 3.15)* | 0.368 |
| **Knee osteoarthritis** |  |  |  |
| MY-NW | Reference | Reference | - |
| MO-NW | 0.95 (0.75 to 1.19) | 0.96 (0.81 to 1.15) | 0.853 |
| MY-OW | 1.8 (1.53 to 2.12)* | 1.83 (1.57 to 2.14)* | 0.969 |
| MO-OW | 1.69 (1.43 to 2.00)* | 1.83 (1.58 to 2.13)* | 0.459 |
| MY-OB | 3.13 (2.64 to 3.72)* | 3.72 (3.17 to 4.36)* | 0.175 |
| MO-OB | 3.08 (2.61 to 3.64)* | 4.03 (3.50 to 4.65)* | **0.013** |
| **Chronic kidney disease** |  |  |  |
| MY-NW | Reference | Reference | - |
| MO-NW | 1.40 (1.18 to 1.66)* | 1.57 (1.33 to 1.86)* | 0.329 |
| MY-OW | 1.27 (1.10 to 1.46)* | 1.39 (1.17 to 1.64)* | 0.420 |
| MO-OW | 1.83 (1.60 to 2.10)* | 2.03 (1.74 to 2.36)* | 0.357 |
| MY-OB | 1.83 (1.57 to 2.13)* | 2.28 (1.90 to 2.73)* | 0.074 |
| MO-OB | 2.84 (2.47 to 3.26)* | 3.30 (2.83 to 3.83)* | 0.185 |
| **Stress urinary incontinence** |  |  |  |
| MY-NW | Reference | Reference | - |
| MO-NW | 0.83 (0.15 to 4.51) | 0.84 (0.60 to 1.17) | 0.946 |
| MY-OW | 2.21 (0.74 to 6.63) | 1.18 (0.87 to 1.61) | 0.305 |
| MO-OW | 1.96 (0.62 to 6.17) | 1.41 (1.06 to 1.88)* | 0.672 |
| MY-OB | 2.20 (0.61 to 7.9) | 1.90 (1.36 to 2.65)* | 0.960 |
| MO-OB | 2.09 (0.6 to 7.23) | 1.68 (1.25 to 2.26)* | 0.969 |
| **Hearing impairment** |  |  |  |
| MY-NW | Reference | Reference | - |
| MO-NW | 1.31 (1.02 to 1.67)* | 1.16 (0.91 to 1.48) | 0.541 |
| MY-OW | 1.13 (0.92 to 1.38) | 1.14 (0.90 to 1.45) | 0.965 |
| MO-OW | 1.14 (0.92 to 1.40) | 1.08 (0.85 to 1.36) | 0.769 |
| MY-OB | 1.09 (0.85 to 1.40) | 1.63 (1.24 to 2.14)* | **0.027** |
| MO-OB | 1.42 (1.14 to 1.77)* | 1.27 (0.99 to 1.63) | 0.616 |
| **Thyroid disorders** |  |  |  |
| MY-NW | Reference | Reference | - |
| MO-NW | 0.97 (0.67 to 1.39) | 1.12 (0.94 to 1.33) | 0.453 |
| MY-OW | 1.23 (0.94 to 1.61) | 1.16 (0.98 to 1.39) | 0.601 |
| MO-OW | 1.25 (0.95 to 1.65) | 1.41 (1.20 to 1.66)* | 0.545 |
| MY-OB | 1.65 (1.22 to 2.24)* | 1.53 (1.24 to 1.87)* | 0.487 |
| MO-OB | 1.70 (1.27 to 2.26)* | 1.85 (1.57 to 2.19)* | 0.799 |
| **Psoriasis** |  |  |  |
| MY-NW | Reference | Reference | - |
| MO-NW | 0.93 (0.65 to 1.32) | 0.93 (0.70 to 1.24) | 0.920 |
| MY-OW | 1.00 (0.77 to 1.30) | 0.96 (0.72 to 1.27) | 0.736 |
| MO-OW | 1.12 (0.85 to 1.47) | 1.04 (0.80 to 1.36) | 0.703 |
| MY-OB | 1.45 (1.07 to 1.95)* | 1.31 (0.95 to 1.81) | 0.672 |
| MO-OB | 1.91 (1.46 to 2.50)* | 1.48 (1.14 to 1.93)* | 0.247 |
| The cox proportional hazard regression was used to estimate the association of metabolomic aging acceleration and body mass index phenotypes with risk of mortality and obesity-related morbidities stratified by sex, adjusting for age, ethnicity, Townsend deprivation index, educational attainment, physical activity, healthy diet, sleep duration, smoking, drinking, and longevity genetic risk scores. Metabolomically younger normal weight was set as the reference group. The asterisk (*) indicates a significant association through two-sided statistical tests. Abbreviations: HR, hazard ratio; CI, confidence interval; MY-NW, metabolomically younger normal weight; MO-NW, metabolomically older normal weight; MY-OW, metabolomically younger overweight; MO-OW, metabolomically older overweight; MY-OB, metabolomically younger obesity; MO-OB, metabolomically older obesity; CVD, cardiovascular disease; COPD, chronic obstructive pulmonary disease. | | | |

| **sTable 3. Associations of metabolomic aging acceleration and body mass index phenotypes with risk of mortality and obesity-related morbidities stratified by age** | | | |
| --- | --- | --- | --- |
| **Mortality and obesity-related morbidities** | **Age < 60** | **Age ≥ 60** | ***p* for interaction** |
|  | **HR (95%CI)** | **HR (95%CI)** |  |
| **All-cause mortality** |  |  |  |
| MY-NW | Reference | Reference | - |
| MO-NW | 1.52 (1.27 to 1.82)* | 1.25 (1.11 to 1.41)* | 0.053 |
| MY-OW | 1.13 (0.95 to 1.34) | 1.01 (0.91 to 1.12) | 0.517 |
| MO-OW | 1.39 (1.18 to 1.64)* | 1.19 (1.07 to 1.32)* | 0.194 |
| MY-OB | 1.31 (1.07 to 1.61)* | 1.16 (1.02 to 1.31)* | 0.506 |
| MO-OB | 1.93 (1.63 to 2.28)* | 1.50 (1.35 to 1.68)* | **0.030** |
| **CVD-specific mortality** |  |  |  |
| MY-NW | Reference | Reference | - |
| MO-NW | 1.41 (0.88 to 2.25) | 1.39 (1.04 to 1.85)* | 0.912 |
| MY-OW | 1.30 (0.85 to 1.98) | 1.05 (0.81 to 1.35) | 0.570 |
| MO-OW | 2.07 (1.39 to 3.08)* | 1.43 (1.12 to 1.83)* | 0.158 |
| MY-OB | 1.65 (1.02 to 2.68)* | 1.48 (1.11 to 1.97)* | 0.855 |
| MO-OB | 3.41 (2.31 to 5.02)* | 2.15 (1.67 to 2.76)* | 0.058 |
| **Cancer-specific mortality** | |  |  |
| MY-NW | Reference | Reference | - |
| MO-NW | 1.38 (1.10 to 1.73)* | 1.14 (0.96 to 1.35) | 0.155 |
| MY-OW | 1.08 (0.86 to 1.34) | 1.03 (0.89 to 1.20) | 0.984 |
| MO-OW | 1.22 (0.98 to 1.51) | 1.20 (1.03 to 1.39)* | 0.941 |
| MY-OB | 1.25 (0.96 to 1.63) | 1.13 (0.95 to 1.36) | 0.766 |
| MO-OB | 1.44 (1.15 to 1.81)* | 1.39 (1.19 to 1.63)* | 0.950 |
| **Coronary heart disease** |  |  |  |
| MY-NW | Reference | Reference | - |
| MO-NW | 1.28 (1.06 to 1.54)* | 1.15 (1.00 to 1.33)* | 0.485 |
| MY-OW | 1.53 (1.31 to 1.79)* | 1.25 (1.11 to 1.40)* | **0.022** |
| MO-OW | 1.74 (1.49 to 2.04)* | 1.41 (1.25 to 1.59)* | **0.028** |
| MY-OB | 2.06 (1.72 to 2.45)* | 1.69 (1.48 to 1.94)* | **0.043** |
| MO-OB | 2.51 (2.15 to 2.94)* | 1.97 (1.74 to 2.23)* | **0.010** |
| **Atrial fibrillation** |  |  |  |
| MY-NW | Reference | Reference | - |
| MO-NW | 1.01 (0.76 to 1.33) | 1.21 (1.03 to 1.40)* | 0.224 |
| MY-OW | 1.14 (0.91 to 1.44) | 1.15 (1.01 to 1.32)* | 0.858 |
| MO-OW | 1.53 (1.22 to 1.91)* | 1.32 (1.16 to 1.50)* | 0.199 |
| MY-OB | 2.36 (1.86 to 3.00)* | 1.71 (1.48 to 1.98)* | **0.013** |
| MO-OB | 2.62 (2.11 to 3.26)* | 2.04 (1.78 to 2.33)* | **0.030** |
| **Heart failure** |  |  |  |
| MY-NW | Reference | Reference | - |
| MO-NW | 1.67 (1.18 to 2.36)* | 1.38 (1.12 to 1.70)* | 0.387 |
| MY-OW | 1.56 (1.14 to 2.13)* | 1.15 (0.96 to 1.38) | 0.118 |
| MO-OW | 1.94 (1.43 to 2.64)* | 1.57 (1.31 to 1.88)* | 0.289 |
| MY-OB | 2.22 (1.57 to 3.13)* | 1.99 (1.64 to 2.42)* | 0.679 |
| MO-OB | 3.81 (2.84 to 5.10)* | 2.98 (2.50 to 3.55)* | 0.217 |
| **Stroke** |  |  |  |
| MY-NW | Reference | Reference | - |
| MO-NW | 1.29 (0.94 to 1.77) | 1.26 (1.02 to 1.55)* | 0.963 |
| MY-OW | 1.17 (0.88 to 1.56) | 1.03 (0.86 to 1.24) | 0.481 |
| MO-OW | 1.29 (0.96 to 1.71) | 1.31 (1.09 to 1.56)* | 0.897 |
| MY-OB | 1.60 (1.15 to 2.22)* | 0.99 (0.79 to 1.25) | **0.016** |
| MO-OB | 1.81 (1.36 to 2.42)* | 1.41 (1.16 to 1.72)* | 0.158 |
| **Peripheral vascular disease** | |  |  |
| MY-NW | Reference | Reference | - |
| MO-NW | 1.24 (0.92 to 1.69) | 1.40 (1.09 to 1.79)* | 0.623 |
| MY-OW | 0.66 (0.48 to 0.91)* | 0.81 (0.64 to 1.02) | 0.185 |
| MO-OW | 1.01 (0.76 to 1.35) | 1.05 (0.83 to 1.31) | 0.673 |
| MY-OB | 0.79 (0.53 to 1.16) | 1.08 (0.82 to 1.42) | 0.119 |
| MO-OB | 1.11 (0.82 to 1.51) | 1.52 (1.20 to 1.92)* | 0.062 |
| **Pulmonary embolism** |  |  |  |
| MY-NW | Reference | Reference | - |
| MO-NW | 1.42 (0.94 to 2.13) | 1.38 (1.00 to 1.90) | 0.915 |
| MY-OW | 1.56 (1.08 to 2.25)* | 1.36 (1.02 to 1.80)* | 0.546 |
| MO-OW | 1.81 (1.26 to 2.59)* | 1.60 (1.21 to 2.12)* | 0.651 |
| MY-OB | 2.55 (1.72 to 3.79)* | 2.26 (1.66 to 3.07)* | 0.716 |
| MO-OB | 2.74 (1.92 to 3.92)* | 2.43 (1.83 to 3.24)* | 0.803 |
| **Aortic valve stenosis** |  |  |  |
| MY-NW | Reference | Reference | - |
| MO-NW | 1.03 (0.52 to 2.06) | 1.30 (0.89 to 1.90) | 0.587 |
| MY-OW | 1.28 (0.72 to 2.28) | 1.44 (1.05 to 1.98)* | 0.694 |
| MO-OW | 1.79 (1.03 to 3.10)* | 1.53 (1.11 to 2.11)* | 0.652 |
| MY-OB | 1.82 (0.96 to 3.46) | 2.14 (1.51 to 3.04)* | 0.617 |
| MO-OB | 3.69 (2.18 to 6.25)* | 3.63 (2.67 to 4.93)* | 0.950 |
| **Lung cancer** |  |  |  |
| MY-NW | Reference | Reference | - |
| MO-NW | 1.31 (0.86 to 2.02) | 0.91 (0.67 to 1.24) | 0.175 |
| MY-OW | 0.94 (0.62 to 1.43) | 0.89 (0.68 to 1.16) | 0.941 |
| MO-OW | 1.15 (0.76 to 1.72) | 0.88 (0.67 to 1.16) | 0.397 |
| MY-OB | 1.03 (0.62 to 1.72) | 0.76 (0.54 to 1.08) | 0.434 |
| MO-OB | 1.08 (0.69 to 1.67) | 0.99 (0.74 to 1.32) | 0.970 |
| **Stomach cancer** |  |  |  |
| MY-NW | Reference | Reference | - |
| MO-NW | 0.68 (0.24 to 1.96) | 1.62 (0.62 to 4.19) | 0.230 |
| MY-OW | 1.00 (0.46 to 2.20) | 2.84 (1.33 to 6.09)* | 0.053 |
| MO-OW | 0.94 (0.41 to 2.15) | 2.65 (1.21 to 5.77)* | 0.059 |
| MY-OB | 0.63 (0.20 to 1.99) | 1.66 (0.65 to 4.22) | 0.176 |
| MO-OB | 1.69 (0.77 to 3.73) | 3.29 (1.47 to 7.34)* | 0.165 |
| **Oesophageal cancer** |  |  |  |
| MY-NW | Reference | Reference | - |
| MO-NW | 1.75 (0.59 to 5.21) | 1.55 (0.80 to 2.99) | 0.897 |
| MY-OW | 2.17 (0.86 to 5.45) | 1.37 (0.77 to 2.41) | 0.332 |
| MO-OW | 1.95 (0.75 to 5.04) | 1.88 (1.08 to 3.28)* | 0.888 |
| MY-OB | 2.35 (0.83 to 6.64) | 1.36 (0.69 to 2.68) | 0.343 |
| MO-OB | 3.59 (1.43 to 9.03)* | 2.05 (1.13 to 3.70)* | 0.287 |
| **Colon cancer** |  |  |  |
| MY-NW | Reference | Reference | - |
| MO-NW | 1.13 (0.71 to 1.79) | 1.64 (1.17 to 2.30)* | 0.189 |
| MY-OW | 1.58 (1.08 to 2.32)* | 1.57 (1.17 to 2.12)* | 0.998 |
| MO-OW | 1.35 (0.90 to 2.01) | 1.82 (1.35 to 2.45)* | 0.219 |
| MY-OB | 1.48 (0.92 to 2.38) | 1.62 (1.13 to 2.31)* | 0.752 |
| MO-OB | 1.67 (1.11 to 2.52)* | 1.69 (1.22 to 2.34)* | 0.932 |
| **Rectal cancer** |  |  |  |
| MY-NW | Reference | Reference | - |
| MO-NW | 1.35 (0.74 to 2.47) | 1.19 (0.73 to 1.96) | 0.700 |
| MY-OW | 0.95 (0.54 to 1.68) | 1.02 (0.67 to 1.56) | 0.829 |
| MO-OW | 1.13 (0.65 to 1.98) | 0.95 (0.61 to 1.48) | 0.590 |
| MY-OB | 0.73 (0.33 to 1.60) | 1.13 (0.68 to 1.89) | 0.362 |
| MO-OB | 1.36 (0.76 to 2.42) | 1.09 (0.68 to 1.77) | 0.497 |
| **Prostate cancer** |  |  |  |
| MY-NW | Reference | Reference | - |
| MO-NW | 1.23 (0.93 to 1.65) | 0.92 (0.73 to 1.15) | 0.138 |
| MY-OW | 1.04 (0.82 to 1.32) | 0.92 (0.78 to 1.09) | 0.338 |
| MO-OW | 0.91 (0.71 to 1.17) | 0.96 (0.80 to 1.14) | 0.728 |
| MY-OB | 0.73 (0.53 to 1.01) | 1.03 (0.83 to 1.26) | 0.108 |
| MO-OB | 0.81 (0.61 to 1.09) | 0.85 (0.69 to 1.05) | 0.904 |
| **Breast cancer** |  |  |  |
| MY-NW | Reference | Reference | - |
| MO-NW | 1.08 (0.88 to 1.31) | 1.00 (0.77 to 1.30) | 0.673 |
| MY-OW | 1.00 (0.81 to 1.24) | 1.24 (0.97 to 1.58) | 0.171 |
| MO-OW | 0.86 (0.70 to 1.07) | 1.17 (0.92 to 1.49) | 0.056 |
| MY-OB | 1.18 (0.91 to 1.53) | 1.39 (1.03 to 1.86)* | 0.330 |
| MO-OB | 1.23 (0.99 to 1.51) | 1.17 (0.90 to 1.52) | 0.907 |
| **Parkinson disease** |  |  |  |
| MY-NW | Reference | Reference | - |
| MO-NW | 1.09 (0.54 to 2.20) | 1.04 (0.71 to 1.51) | 0.859 |
| MY-OW | 1.64 (0.92 to 2.92) | 0.98 (0.72 to 1.35) | 0.197 |
| MO-OW | 0.71 (0.35 to 1.44) | 1.18 (0.86 to 1.61) | 0.153 |
| MY-OB | 1.17 (0.55 to 2.49) | 1.00 (0.68 to 1.48) | 0.821 |
| MO-OB | 1.47 (0.78 to 2.76) | 1.02 (0.71 to 1.46) | 0.342 |
| **Dementia** |  |  |  |
| MY-NW | Reference | Reference | - |
| MO-NW | 1.53 (0.81 to 2.87) | 1.20 (0.96 to 1.49) | 0.572 |
| MY-OW | 1.06 (0.59 to 1.92) | 0.79 (0.65 to 0.97)* | 0.280 |
| MO-OW | 1.22 (0.68 to 2.18) | 0.83 (0.68 to 1.02) | 0.200 |
| MY-OB | 1.23 (0.62 to 2.44) | 0.80 (0.61 to 1.03) | 0.131 |
| MO-OB | 1.84 (1.05 to 3.24)* | 1.00 (0.80 to 1.25) | **0.013** |
| **Epilepsy** |  |  |  |
| MY-NW | Reference | Reference | - |
| MO-NW | 0.81 (0.48 to 1.37) | 1.07 (0.70 to 1.65) | 0.458 |
| MY-OW | 1.28 (0.84 to 1.95) | 1.03 (0.71 to 1.50) | 0.448 |
| MO-OW | 1.26 (0.83 to 1.93) | 0.75 (0.50 to 1.13) | 0.069 |
| MY-OB | 1.34 (0.81 to 2.24) | 0.93 (0.57 to 1.50) | 0.239 |
| MO-OB | 1.38 (0.89 to 2.15) | 1.26 (0.84 to 1.89) | 0.580 |
| **Depression** |  |  |  |
| MY-NW | Reference | Reference | - |
| MO-NW | 0.97 (0.79 to 1.19) | 1.13 (0.91 to 1.39) | 0.333 |
| MY-OW | 1.36 (1.14 to 1.63)* | 1.08 (0.89 to 1.31) | 0.127 |
| MO-OW | 1.55 (1.30 to 1.84)* | 1.02 (0.84 to 1.24) | **0.003** |
| MY-OB | 1.81 (1.48 to 2.22)* | 1.29 (1.03 to 1.62)* | **0.028** |
| MO-OB | 2.40 (2.03 to 2.84)* | 1.42 (1.16 to 1.73)* | **<0.001** |
| **Anxiety** |  |  |  |
| MY-NW | Reference | Reference | - |
| MO-NW | 0.90 (0.76 to 1.07) | 1.13 (0.94 to 1.37) | 0.092 |
| MY-OW | 1.10 (0.95 to 1.29) | 1.12 (0.95 to 1.33) | 0.971 |
| MO-OW | 1.16 (1.00 to 1.36) | 1.26 (1.07 to 1.49)* | 0.589 |
| MY-OB | 1.42 (1.19 to 1.71)* | 1.34 (1.10 to 1.64)* | 0.442 |
| MO-OB | 1.68 (1.44 to 1.95)* | 1.15 (0.95 to 1.38) | **<0.001** |
| **Dyspepsia** |  |  |  |
| MY-NW | Reference | Reference | - |
| MO-NW | 1.15 (1.04 to 1.28)* | 1.15 (1.04 to 1.27)* | 0.840 |
| MY-OW | 1.21 (1.10 to 1.33)* | 1.18 (1.07 to 1.29)* | 0.958 |
| MO-OW | 1.27 (1.16 to 1.39)* | 1.24 (1.14 to 1.36)* | 0.894 |
| MY-OB | 1.45 (1.30 to 1.62)* | 1.29 (1.15 to 1.44)* | 0.233 |
| MO-OB | 1.65 (1.50 to 1.81)* | 1.31 (1.18 to 1.44)* | **0.001** |
| **Gastroesophageal reflux disease** |  |  |  |
| MY-NW | Reference | Reference | - |
| MO-NW | 1.06 (0.92 to 1.22) | 1.24 (1.08 to 1.42)* | 0.141 |
| MY-OW | 1.33 (1.18 to 1.51)* | 1.33 (1.18 to 1.51)* | 0.838 |
| MO-OW | 1.42 (1.25 to 1.60)* | 1.45 (1.28 to 1.63)* | 0.767 |
| MY-OB | 1.68 (1.46 to 1.94)* | 1.56 (1.36 to 1.81)* | 0.485 |
| MO-OB | 1.89 (1.67 to 2.14)* | 1.58 (1.38 to 1.80)* | **0.038** |
| **Cholelithiasis** |  |  |  |
| MY-NW | Reference | Reference | - |
| MO-NW | 0.95 (0.73 to 1.24) | 1.37 (1.06 to 1.77)* | 0.076 |
| MY-OW | 2.18 (1.77 to 2.69)* | 2.06 (1.66 to 2.55)* | 0.810 |
| MO-OW | 1.94 (1.57 to 2.40)* | 1.95 (1.57 to 2.42)* | 0.707 |
| MY-OB | 3.80 (3.05 to 4.73)* | 2.92 (2.31 to 3.69)* | 0.229 |
| MO-OB | 4.05 (3.32 to 4.94)* | 3.11 (2.50 to 3.87)* | 0.107 |
| **Cholecystitis** |  |  |  |
| MY-NW | Reference | Reference | - |
| MO-NW | 1.13 (0.63 to 2.00) | 1.37 (0.65 to 2.93) | 0.765 |
| MY-OW | 1.52 (0.92 to 2.52) | 2.19 (1.17 to 4.07)* | 0.207 |
| MO-OW | 1.30 (0.77 to 2.20) | 2.58 (1.40 to 4.78)* | 0.064 |
| MY-OB | 2.55 (1.48 to 4.4)* | 3.51 (1.82 to 6.76)* | 0.288 |
| MO-OB | 3.00 (1.87 to 4.83)* | 3.12 (1.65 to 5.89)* | 0.802 |
| **Nonalcoholic fatty liver disease** | |  |  |
| MY-NW | Reference | Reference | - |
| MO-NW | 1.64 (0.96 to 2.81) | 1.83 (0.95 to 3.53) | 0.807 |
| MY-OW | 3.51 (2.26 to 5.48)* | 3.16 (1.81 to 5.51)* | 0.829 |
| MO-OW | 3.73 (2.39 to 5.80)* | 4.32 (2.50 to 7.46)* | 0.616 |
| MY-OB | 7.17 (4.57 to 11.23)* | 6.66 (3.79 to 11.70)* | 0.930 |
| MO-OB | 11.09 (7.31 to 16.83)* | 8.20 (4.78 to 14.07)* | 0.455 |
| **Constipation** |  |  |  |
| MY-NW | Reference | Reference | - |
| MO-NW | 1.07 (0.89 to 1.28) | 1.02 (0.87 to 1.19) | 0.551 |
| MY-OW | 1.01 (0.85 to 1.20) | 0.99 (0.87 to 1.13) | 0.564 |
| MO-OW | 1.16 (0.99 to 1.37) | 1.11 (0.97 to 1.27) | 0.938 |
| MY-OB | 1.29 (1.06 to 1.58)* | 1.20 (1.03 to 1.40)* | 0.982 |
| MO-OB | 1.28 (1.08 to 1.53)* | 1.16 (1.00 to 1.34)* | 0.634 |
| **Chronic liver disease** |  |  |  |
| MY-NW | Reference | Reference | - |
| MO-NW | 1.95 (0.78 to 4.86) | 1.32 (0.59 to 2.95) | 0.519 |
| MY-OW | 1.39 (0.57 to 3.37) | 1.19 (0.59 to 2.41) | 0.724 |
| MO-OW | 2.47 (1.10 to 5.58)* | 1.62 (0.82 to 3.20) | 0.375 |
| MY-OB | 1.41 (0.49 to 4.10) | 0.89 (0.35 to 2.28) | 0.461 |
| MO-OB | 3.72 (1.67 to 8.30)* | 2.44 (1.23 to 4.87)* | 0.343 |
| **Diverticular disease** |  |  |  |
| MY-NW | Reference | Reference | - |
| MO-NW | 1.15 (1.01 to 1.31)* | 1.16 (1.03 to 1.29)* | 0.932 |
| MY-OW | 1.46 (1.31 to 1.63)* | 1.19 (1.08 to 1.31)* | **0.004** |
| MO-OW | 1.43 (1.28 to 1.60)* | 1.36 (1.24 to 1.50)* | 0.458 |
| MY-OB | 1.86 (1.63 to 2.11)* | 1.41 (1.25 to 1.58)* | **0.001** |
| MO-OB | 1.95 (1.74 to 2.19)* | 1.56 (1.41 to 1.73)* | **0.004** |
| **Inflammatory bowel disease** | |  |  |
| MY-NW | Reference | Reference | - |
| MO-NW | 1.03 (0.66 to 1.60) | 0.75 (0.45 to 1.24) | 0.345 |
| MY-OW | 0.82 (0.54 to 1.24) | 0.90 (0.60 to 1.35) | 0.794 |
| MO-OW | 1.00 (0.67 to 1.49) | 1.08 (0.73 to 1.61) | 0.863 |
| MY-OB | 1.05 (0.64 to 1.71) | 0.88 (0.53 to 1.46) | 0.557 |
| MO-OB | 1.08 (0.71 to 1.65) | 1.02 (0.65 to 1.58) | 0.699 |
| **Irritable bowel syndrome** |  |  |  |
| MY-NW | Reference | Reference | - |
| MO-NW | 0.96 (0.73 to 1.27) | 1.32 (0.97 to 1.79) | 0.160 |
| MY-OW | 0.98 (0.76 to 1.28) | 1.21 (0.91 to 1.61) | 0.229 |
| MO-OW | 1.18 (0.92 to 1.52) | 1.17 (0.88 to 1.56) | 0.996 |
| MY-OB | 1.38 (1.02 to 1.86)* | 1.19 (0.84 to 1.70) | 0.624 |
| MO-OB | 1.52 (1.19 to 1.96)* | 0.94 (0.67 to 1.31) | **0.023** |
| **Asthma** |  |  |  |
| MY-NW | Reference | Reference | - |
| MO-NW | 1.23 (0.98 to 1.54) | 1.04 (0.81 to 1.34) | 0.313 |
| MY-OW | 1.25 (1.02 to 1.54)* | 1.22 (0.98 to 1.51) | 0.976 |
| MO-OW | 1.26 (1.02 to 1.55)* | 1.29 (1.04 to 1.59)* | 0.801 |
| MY-OB | 1.52 (1.19 to 1.95)* | 1.60 (1.25 to 2.04)* | 0.619 |
| MO-OB | 2.05 (1.67 to 2.51)* | 2.02 (1.63 to 2.51)* | 0.889 |
| **COPD** |  |  |  |
| MY-NW | Reference | Reference | - |
| MO-NW | 1.31 (1.00 to 1.70) | 1.28 (1.07 to 1.53)* | 0.913 |
| MY-OW | 1.23 (0.96 to 1.56) | 0.89 (0.76 to 1.05) | **0.043** |
| MO-OW | 1.35 (1.06 to 1.71)* | 1.11 (0.95 to 1.30) | 0.218 |
| MY-OB | 1.46 (1.1 to 1.92)* | 1.20 (1.00 to 1.43) | 0.272 |
| MO-OB | 1.99 (1.57 to 2.51)* | 1.41 (1.20 to 1.66)* | **0.020** |
| **Bronchiectasis** |  |  |  |
| MY-NW | Reference | Reference | - |
| MO-NW | 1.05 (0.67 to 1.65) | 1.21 (0.89 to 1.66) | 0.664 |
| MY-OW | 0.83 (0.53 to 1.28) | 0.82 (0.61 to 1.10) | 0.770 |
| MO-OW | 0.76 (0.49 to 1.20) | 1.08 (0.82 to 1.43) | 0.124 |
| MY-OB | 0.87 (0.51 to 1.50) | 0.77 (0.53 to 1.12) | 0.922 |
| MO-OB | 0.98 (0.63 to 1.54) | 0.87 (0.63 to 1.21) | 0.874 |
| **Sleep apnea** |  |  |  |
| MY-NW | Reference | Reference | - |
| MO-NW | 1.11 (0.65 to 1.92) | 1.08 (0.62 to 1.88) | 0.869 |
| MY-OW | 2.54 (1.68 to 3.84)* | 1.65 (1.08 to 2.53)* | 0.178 |
| MO-OW | 3.02 (2.01 to 4.55)* | 2.15 (1.41 to 3.28)* | 0.278 |
| MY-OB | 9.15 (6.15 to 13.60)* | 5.55 (3.67 to 8.38)* | 0.077 |
| MO-OB | 12.31 (8.44 to 17.97)* | 7.43 (5.01 to 11.01)* | 0.057 |
| **Cataract** |  |  |  |
| MY-NW | Reference | Reference | - |
| MO-NW | 1.05 (0.89 to 1.23) | 1.05 (0.96 to 1.16) | 0.850 |
| MY-OW | 1.11 (0.96 to 1.29) | 1.14 (1.05 to 1.25)* | 0.934 |
| MO-OW | 1.24 (1.07 to 1.43)* | 1.14 (1.04 to 1.24)* | 0.231 |
| MY-OB | 1.22 (1.02 to 1.46)* | 1.15 (1.03 to 1.28)* | 0.375 |
| MO-OB | 1.41 (1.21 to 1.64)* | 1.31 (1.19 to 1.43)* | 0.296 |
| **Glaucoma** |  |  |  |
| MY-NW | Reference | Reference | - |
| MO-NW | 0.78 (0.57 to 1.08) | 1.13 (0.91 to 1.40) | 0.073 |
| MY-OW | 0.92 (0.7 to 1.22) | 1.00 (0.83 to 1.21) | 0.718 |
| MO-OW | 0.74 (0.55 to 1.00)* | 1.02 (0.84 to 1.23) | 0.112 |
| MY-OB | 0.80 (0.55 to 1.15) | 0.91 (0.71 to 1.16) | 0.722 |
| MO-OB | 1.02 (0.76 to 1.38) | 0.96 (0.77 to 1.19) | 0.480 |
| **AMD** |  |  |  |
| MY-NW | Reference | Reference | - |
| MO-NW | 0.83 (0.55 to 1.24) | 1.15 (0.91 to 1.47) | 0.166 |
| MY-OW | 0.85 (0.59 to 1.23) | 1.12 (0.90 to 1.39) | 0.226 |
| MO-OW | 0.81 (0.56 to 1.18) | 1.29 (1.05 to 1.59)* | **0.036** |
| MY-OB | 1.08 (0.70 to 1.68) | 1.27 (0.98 to 1.65) | 0.580 |
| MO-OB | 0.96 (0.65 to 1.43) | 1.53 (1.22 to 1.91)* | 0.051 |
| **Osteoporosis** |  |  |  |
| MY-NW | Reference | Reference | - |
| MO-NW | 1.03 (0.87 to 1.21) | 0.99 (0.86 to 1.15) | 0.743 |
| MY-OW | 1.72 (1.49 to 1.98)* | 1.52 (1.35 to 1.72)* | 0.178 |
| MO-OW | 1.72 (1.49 to 1.98)* | 1.47 (1.30 to 1.66)* | 0.079 |
| MY-OB | 2.65 (2.27 to 3.10)* | 2.67 (2.34 to 3.05)* | 0.937 |
| MO-OB | 3.03 (2.64 to 3.47)* | 2.48 (2.19 to 2.81)* | **0.022** |
| **Knee osteoarthritis** |  |  |  |
| MY-NW | Reference | Reference | - |
| MO-NW | 0.86 (0.69 to 1.06) | 1.03 (0.85 to 1.23) | 0.235 |
| MY-OW | 1.88 (1.59 to 2.23)* | 1.86 (1.61 to 2.15)* | 0.859 |
| MO-OW | 1.70 (1.43 to 2.02)* | 1.85 (1.59 to 2.14)* | 0.558 |
| MY-OB | 3.26 (2.72 to 3.89)* | 3.66 (3.14 to 4.27)* | 0.437 |
| MO-OB | 3.97 (3.39 to 4.66)* | 3.36 (2.90 to 3.89)* | 0.070 |
| **Chronic kidney disease** |  |  |  |
| MY-NW | Reference | Reference | - |
| MO-NW | 1.59 (1.26 to 2.00)* | 1.45 (1.26 to 1.66)* | 0.486 |
| MY-OW | 1.62 (1.32 to 1.99)* | 1.23 (1.09 to 1.39)* | **0.037** |
| MO-OW | 2.36 (1.94 to 2.87)* | 1.78 (1.58 to 2.01)* | **0.023** |
| MY-OB | 2.44 (1.95 to 3.05)* | 1.86 (1.62 to 2.13)* | 0.051 |
| MO-OB | 3.83 (3.16 to 4.64)* | 2.77 (2.46 to 3.12)* | **0.005** |
| **Stress urinary incontinence** | |  |  |
| MY-NW | Reference | Reference | - |
| MO-NW | 1.09 (0.73 to 1.63) | 0.52 (0.29 to 0.93)* | **0.036** |
| MY-OW | 1.26 (0.85 to 1.85) | 1.19 (0.77 to 1.83) | 0.983 |
| MO-OW | 1.93 (1.36 to 2.75)* | 0.91 (0.58 to 1.43) | **0.012** |
| MY-OB | 1.73 (1.11 to 2.70)* | 1.95 (1.22 to 3.13)* | 0.645 |
| MO-OB | 1.87 (1.29 to 2.71)* | 1.45 (0.92 to 2.27) | 0.361 |
| **Hearing impairment** |  |  |  |
| MY-NW | Reference | Reference | - |
| MO-NW | 1.13 (0.82 to 1.56) | 1.27 (1.03 to 1.56)* | 0.584 |
| MY-OW | 1.07 (0.80 to 1.44) | 1.15 (0.96 to 1.37) | 0.639 |
| MO-OW | 1.19 (0.89 to 1.58) | 1.08 (0.90 to 1.30) | 0.602 |
| MY-OB | 1.17 (0.82 to 1.66) | 1.31 (1.05 to 1.62)* | 0.603 |
| MO-OB | 1.54 (1.15 to 2.07)* | 1.27 (1.04 to 1.55)* | 0.234 |
| **Thyroid disorders** |  |  |  |
| MY-NW | Reference | Reference | - |
| MO-NW | 1.08 (0.86 to 1.35) | 1.10 (0.88 to 1.38) | 0.917 |
| MY-OW | 1.17 (0.95 to 1.44) | 1.21 (0.99 to 1.47) | 0.667 |
| MO-OW | 1.50 (1.23 to 1.83)* | 1.24 (1.02 to 1.52)* | 0.235 |
| MY-OB | 1.53 (1.19 to 1.95)* | 1.62 (1.29 to 2.04)* | 0.564 |
| MO-OB | 1.96 (1.6 to 2.39)* | 1.68 (1.37 to 2.07)* | 0.386 |
| **Psoriasis** |  |  |  |
| MY-NW | Reference | Reference | - |
| MO-NW | 1.07 (0.76 to 1.49) | 0.84 (0.63 to 1.12) | 0.254 |
| MY-OW | 1.15 (0.85 to 1.55) | 0.84 (0.66 to 1.07) | 0.218 |
| MO-OW | 1.34 (1.00 to 1.79) | 0.89 (0.69 to 1.13) | 0.064 |
| MY-OB | 1.84 (1.33 to 2.55)* | 1.08 (0.81 to 1.43) | **0.032** |
| MO-OB | 2.29 (1.73 to 3.03)* | 1.29 (1.00 to 1.65)* | **0.006** |
| The cox proportional hazard regression was used to estimate the association of metabolomic aging acceleration and body mass index phenotypes with risk of mortality and obesity-related morbidities stratified by age group, adjusting for age, sex, ethnicity, Townsend deprivation index, educational attainment, physical activity, healthy diet, sleep duration, smoking, drinking, and longevity genetic risk scores. Metabolomically younger normal weight was set as the reference group. The asterisk (*) indicates a significant association through two-sided statistical tests.  Abbreviations: HR, hazard ratio; CI, confidence interval; MY-NW, metabolomically younger normal weight; MO-NW, metabolomically older normal weight; MY-OW, metabolomically younger overweight; MO-OW, metabolomically older overweight; MY-OB, metabolomically younger obesity; MO-OB, metabolomically older obesity; CVD, cardiovascular disease; COPD, chronic obstructive pulmonary disease. | | | |


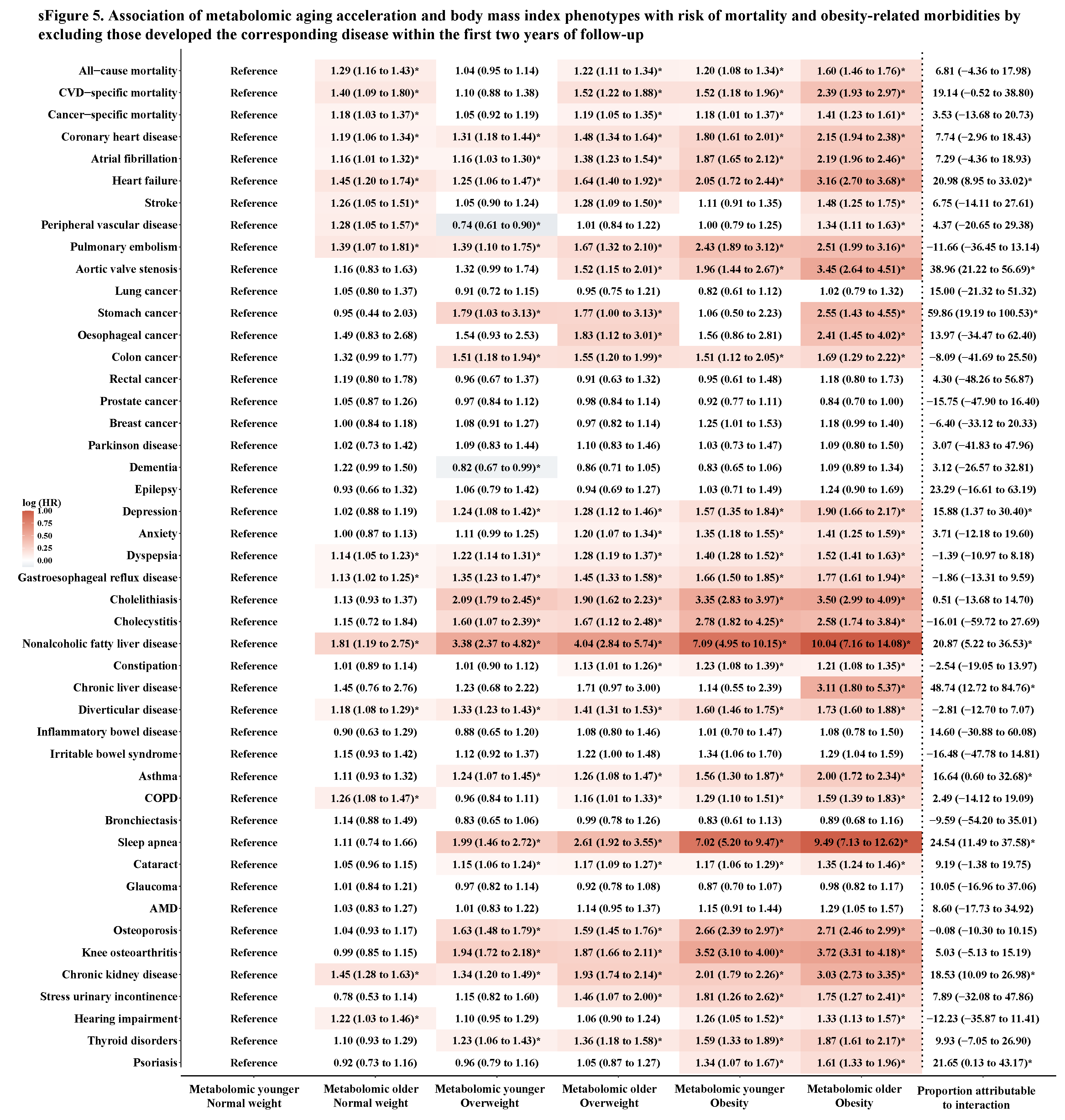


**sFigure 5. Association of metabolomic aging acceleration and body mass index phenotypes with risk of mortality and obesity-related morbidities by excluding those developed the corresponding disease within the first two years of follow-up**

The cox proportional hazard regression was used to estimate the association of metabolomic aging acceleration and body mass index phenotypes with risk of mortality and obesity-related morbidities by excluding those developed the corresponding disease within the first two years of follow-up, adjusting for age, sex, ethnicity, Townsend deprivation index, educational attainment, physical activity, healthy diet, sleep duration, smoking, drinking, and longevity genetic risk scores. Metabolomic younger normal weight was set as the reference group. Prostate cancer was analyzed only in males. Breast cancer was analyzed only in females. Proportion attributable due to interaction and corresponding 95% confidence intervals was used as the measure of additive interaction between the metabolomic aging acceleration (younger vs older) and obesity status (normal weight vs obesity), and the additive interaction was statistically significant when its confidence interval did not include 0. The asterisk (*) indicates a significant association through two-sided statistical tests.

Abbreviations: HR, hazard ratio; CVD, cardiovascular disease; COPD, chronic obstructive pulmonary disease; AMD, age-related macular degeneration.

**
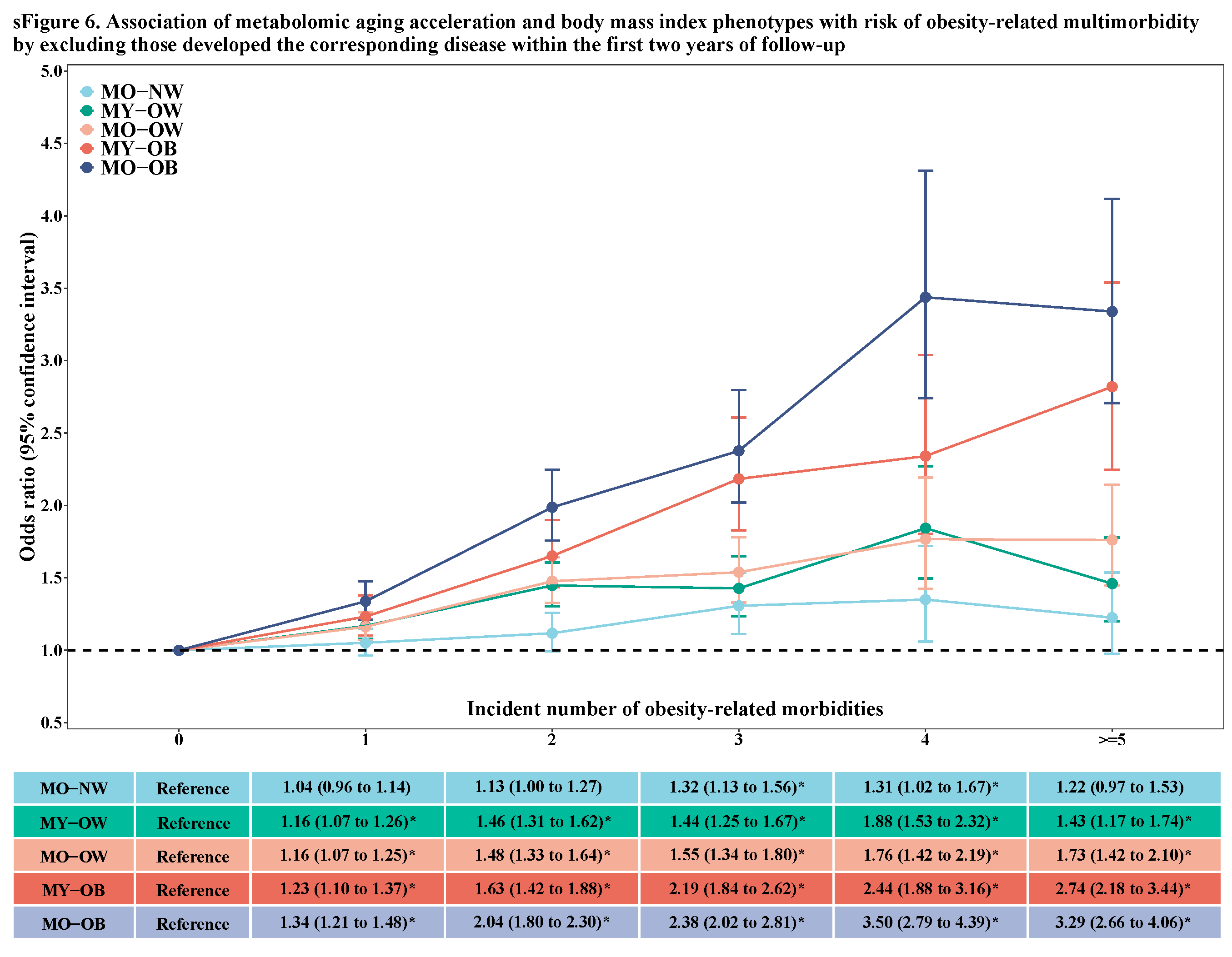
**

**sFigure 6. Association of metabolomic aging acceleration and body mass index phenotypes with risk of obesity-related multimorbidity by excluding those developed the corresponding disease within the first two years of follow-up**

Multinomial logistic regression was used to estimate the associations of metabolomic aging acceleration and body mass index phenotypes with risk of obesity-related multimorbidity by excluding those developed the corresponding disease within the first two years of follow-up, adjusting for age, sex, ethnicity, Townsend deprivation index, educational attainment, physical activity, healthy diet, sleep duration, smoking, drinking, and longevity genetic risk scores. Metabolomic younger normal weight was set as the reference group. The asterisk (*) indicates a significant association through two-sided statistical tests.

Abbreviations: MO-NW, metabolomically older normal weight; MY-OW, metabolomically younger overweight; MO-OW, metabolomically older overweight; MY-OB, metabolomically younger obesity; MO-OB, metabolomically older obesity.

**
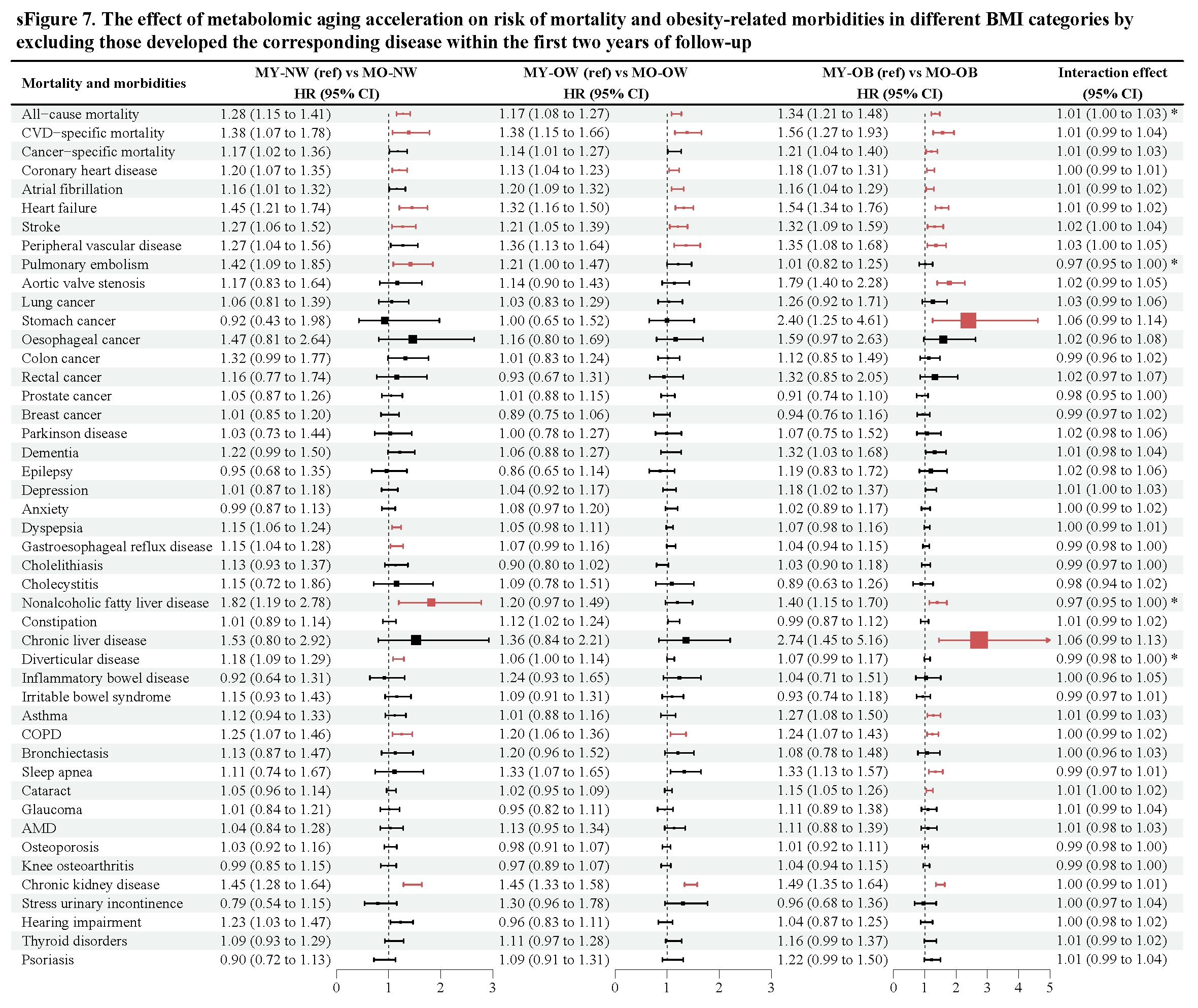
**

**sFigure 7. The effect of metabolomic aging acceleration on risk of mortality and obesity-related morbidities in different BMI categories by excluding those developed the corresponding disease within the first two years of follow-up**

The cox proportional hazard regression was used to estimate the effect of metabolomic aging acceleration on risk of mortality and obesity-related morbidities in different BMI categories by excluding those developed the corresponding disease within the first two years of follow-up, adjusting for age, sex, ethnicity, Townsend deprivation index, educational attainment, physical activity, healthy diet, sleep duration, smoking, drinking, and longevity genetic risk scores. Multiplicative interaction was evaluated using hazard ratios for the product term between the metabolomic aging status (younger or older) and body mass index (continuous). Prostate cancer was analyzed only in males. Breast cancer was analyzed only in females. Squares indicate the hazard ratios, with black color denoting non-significant association, red color indicating positive association, and blue color indicating inverse association.

Abbreviations: BMI, body mass index; MY-NW, metabolomically younger normal weight; MO-NW, metabolomically older normal weight; MY-OW, metabolomically younger overweight; MO-OW, metabolomically older overweight; MY-OB, metabolomically younger obesity; MO-OB, metabolomically older obesity; HR, hazard ratio; CI, confidence interval; CVD, cardiovascular disease; COPD, chronic obstructive pulmonary disease; AMD, age-related macular degeneration.

| **sTable 4. The mediating role of the INFLA-score in the association between metabolomically younger overweight/obesity phenotypes and risk of mortality and obesity-related morbidities by excluding those developed the corresponding disease within the first two years of follow-up** | | | | |
| --- | --- | --- | --- | --- |
| **Mortality and obesity-related morbidities** | **Total effect** | **Direct effect** | **Mediation effect** | **Proportion mediated** |
| **Metabolomically younger overweight** | | | | |
| Coronary heart disease | 0.24 (0.11 to 0.37)* | 0.21 (0.08 to 0.34)* | 0.06 (0.03 to 0.08)* | 23.98 (7.06 to 40.89)* |
| Atrial fibrillation | 0.13 (-0.01 to 0.27) | 0.15 (0.01 to 0.29)* | -0.01 (-0.04 to 0.01) | -10.14 (-33.43 to 13.15) |
| Heart failure | 0.21 (0.00 to 0.43) | 0.21 (0.00 to 0.43) | 0.06 (0.02 to 0.10)* | 29.67 (-5.13 to 64.48) |
| Pulmonary embolism | 0.37 (0.02 to 0.72)* | 0.34 (-0.01 to 0.68) | -0.01 (-0.07 to 0.05) | -2.87 (-18.38 to 12.65) |
| Stomach cancer | 0.63 (-0.32 to 1.58) | 0.68 (-0.29 to 1.65) | 0.13 (-0.02 to 0.28) | 20.81 (-18.63 to 60.25) |
| Colon cancer | 0.63 (0.19 to 1.06)* | 0.65 (0.21 to 1.10)* | 0.06 (0.00 to 0.12) | 9.55 (-2.68 to 21.78) |
| Depression | 0.23 (0.05 to 0.41)* | 0.22 (0.04 to 0.40)* | 0.05 (0.01 to 0.08)* | 19.46 (-0.43 to 39.36) |
| Dyspepsia | 0.19 (0.10 to 0.27)* | 0.17 (0.08 to 0.26)* | 0.02 (0.00 to 0.04)* | 10.69 (0.53 to 20.85)* |
| Gastroesophageal reflux disease | 0.31 (0.18 to 0.43)* | 0.29 (0.16 to 0.41)* | 0.03 (0.01 to 0.05)* | 9.23 (1.22 to 17.23)* |
| Cholelithiasis | 0.99 (0.65 to 1.33)* | 0.95 (0.62 to 1.29)* | 0.08 (0.04 to 0.12)* | 8.39 (3.26 to 13.52)* |
| Cholecystitis | 0.50 (-0.15 to 1.14) | 0.32 (-0.27 to 0.92) | 0.05 (-0.05 to 0.15) | 10.38 (-13.73 to 34.49) |
| Nonalcoholic fatty liver disease | 1.90 (0.81 to 2.99)* | 1.79 (0.73 to 2.85)* | 0.07 (-0.02 to 0.17) | 3.85 (-1.68 to 9.39) |
| Diverticular disease | 0.33 (0.22 to 0.44)* | 0.30 (0.19 to 0.41)* | 0.04 (0.03 to 0.06)* | 13.63 (6.18 to 21.08)* |
| Asthma | 0.26 (0.05 to 0.47)* | 0.23 (0.02 to 0.44)* | 0.04 (0.01 to 0.08)* | 16.30 (-3.28 to 35.88) |
| Sleep apnea | 0.93 (0.28 to 1.59)* | 0.91 (0.26 to 1.55)* | 0.01 (-0.07 to 0.09) | 1.04 (-7.65 to 9.72) |
| Cataract | 0.17 (0.07 to 0.26)* | 0.15 (0.06 to 0.25)* | 0.01 (-0.01 to 0.03) | 4.81 (-6.35 to 15.96) |
| Osteoporosis | 0.59 (0.43 to 0.76)* | 0.60 (0.43 to 0.76)* | 0.02 (0.00 to 0.04) | 3.13 (-0.95 to 7.22) |
| Knee osteoarthritis | 0.85 (0.62 to 1.09)* | 0.85 (0.62 to 1.09)* | 0.02 (-0.01 to 0.05) | 2.78 (-0.83 to 6.38) |
| Chronic kidney disease | 0.31 (0.15 to 0.46)* | 0.27 (0.12 to 0.42)* | 0.06 (0.03 to 0.09)* | 20.15 (6.88 to 33.42)* |
| Thyroid disorders | 0.21 (0.01 to 0.41)* | 0.19 (0.00 to 0.39) | 0.03 (0.00 to 0.07) | 15.17 (-6.68 to 37.02) |
| **Metabolomically younger obesity** | |  |  |  |
| All-cause mortality | 0.21 (0.06 to 0.36)* | 0.14 (-0.01 to 0.30) | 0.16 (0.11 to 0.21)* | 75.71 (19.26 to 132.17)* |
| CVD-specific mortality | 0.66 (0.2 to 1.12)* | 0.52 (0.06 to 0.98)* | 0.23 (0.10 to 0.37)* | 35.42 (4.83 to 66.01)* |
| Cancer-specific mortality | 0.2 (0.00 to 0.41) | 0.17 (-0.05 to 0.38) | 0.14 (0.07 to 0.21)* | 67.94 (-8.11 to 143.98) |
| Coronary heart disease | 0.75 (0.54 to 0.97)* | 0.72 (0.50 to 0.95)* | 0.12 (0.07 to 0.18)* | 16.37 (7.64 to 25.10)* |
| Atrial fibrillation | 0.92 (0.65 to 1.18)* | 0.95 (0.67 to 1.23)* | -0.02 (-0.08 to 0.03) | -2.58 (-8.77 to 3.60) |
| Heart failure | 1.11 (0.70 to 1.52)* | 1.00 (0.59 to 1.41)* | 0.15 (0.05 to 0.24)* | 13.09 (3.62 to 22.56)* |
| Pulmonary embolism | 1.58 (0.85 to 2.30)* | 1.66 (0.89 to 2.44)* | 0.00 (-0.12 to 0.12) | -0.06 (-7.61 to 7.49) |
| Venous thromboembolism | 0.95 (0.25 to 1.65)* | 0.92 (0.19 to 1.64)* | 0.13 (-0.04 to 0.30) | 13.71 (-6.38 to 33.8) |
| Aortic valve stenosis | 0.92 (0.26 to 1.58)* | 0.68 (0.06 to 1.3)* | 0.05 (-0.10 to 0.21) | 5.88 (-11.00 to 22.75) |
| Colon cancer | 0.44 (-0.05 to 0.93) | 0.57 (0.03 to 1.1)* | 0.14 (-0.01 to 0.28) | 30.88 (-16.04 to 77.79) |
| Depression | 0.66 (0.37 to 0.94)* | 0.60 (0.30 to 0.90)* | 0.11 (0.04 to 0.18)* | 16.62 (4.05 to 29.19)* |
| Anxiety | 0.38 (0.17 to 0.59)* | 0.35 (0.13 to 0.57)* | 0.06 (0.00 to 0.11)* | 14.88 (-1.98 to 31.75) |
| Dyspepsia | 0.34 (0.21 to 0.46)* | 0.32 (0.19 to 0.45)* | 0.04 (0.01 to 0.08)* | 12.20 (1.02 to 23.38)* |
| Gastroesophageal reflux disease | 0.59 (0.40 to 0.77)* | 0.56 (0.36 to 0.75)* | 0.06 (0.01 to 0.10)* | 9.84 (1.46 to 18.23)* |
| Cholelithiasis | 2.27 (1.66 to 2.87)* | 1.9 (1.31 to 2.48)* | 0.18 (0.09 to 0.28)* | 7.95 (3.30 to 12.60)* |
| Cholecystitis | 1.64 (0.40 to 2.88)* | 1.49 (0.23 to 2.75)* | 0.11 (-0.11 to 0.33) | 6.73 (-7.42 to 20.87) |
| Nonalcoholic fatty liver disease | 5.30 (2.89 to 7.71)* | 4.68 (2.40 to 6.97)* | 0.16 (-0.05 to 0.38) | 3.08 (-1.25 to 7.40) |
| Constipation | 0.16 (-0.01 to 0.32) | 0.12 (-0.05 to 0.29) | 0.03 (-0.02 to 0.08) | 21.03 (-18.54 to 60.61) |
| Diverticular disease | 0.58 (0.42 to 0.73)* | 0.54 (0.38 to 0.71)* | 0.10 (0.06 to 0.14)* | 17.29 (8.59 to 25.99)* |
| Asthma | 0.56 (0.24 to 0.87)* | 0.45 (0.14 to 0.77)* | 0.10 (0.02 to 0.18)* | 18.43 (0.66 to 36.20)* |
| COPD | 0.32 (0.08 to 0.55)* | 0.1 (-0.13 to 0.32) | 0.24 (0.16 to 0.32)* | 75.65 (18.35 to 132.94)* |
| Sleep apnea | 5.82 (3.58 to 8.07)* | 4.64 (2.70 to 6.57)* | 0.01 (-0.16 to 0.17) | 0.09 (-2.76 to 2.94) |
| Cataract | 0.17 (0.05 to 0.3)* | 0.15 (0.02 to 0.28)* | 0.01 (-0.02 to 0.05) | 8.31 (-14.07 to 30.70) |
| Osteoporosis | 1.66 (1.35 to 1.97)* | 1.7 (1.37 to 2.03)* | 0.03 (-0.01 to 0.08) | 2.09 (-0.93 to 5.12) |
| Knee osteoarthritis | 2.38 (1.91 to 2.84)* | 2.4 (1.91 to 2.88)* | 0.05 (-0.01 to 0.11) | 2.06 (-0.64 to 4.77) |
| Chronic kidney disease | 1.06 (0.79 to 1.33)* | 0.99 (0.72 to 1.26)* | 0.14 (0.08 to 0.21)* | 13.62 (6.91 to 20.32)* |
| Stress urinary incontinence | 1.10 (0.24 to 1.95)* | 1.32 (0.36 to 2.28)* | 0.05 (-0.11 to 0.21) | 4.49 (-10.27 to 19.25) |
| Hearing impairment | 0.31 (0.04 to 0.58)* | 0.32 (0.03 to 0.60)* | 0.05 (-0.03 to 0.12) | 14.73 (-13.95 to 43.42) |
| Thyroid disorders | 0.59 (0.28 to 0.90)* | 0.47 (0.16 to 0.79)* | 0.07 (0.00 to 0.15) | 12.40 (-1.67 to 26.48) |
| Psoriasis | 0.32 (0.00 to 0.64) | 0.26 (-0.07 to 0.59) | 0.07 (-0.02 to 0.17) | 22.37 (-14.61 to 59.35) |
| Total effect indicated the effect of the metabolomically younger overweight/obesity phenotypes (MY-O phenotypes) on the outcomes. Direct effect indicated the effect of the MY-O phenotypes on the outcomes. Mediation effect indicated the effect of the MY-O phenotypes on the outcomes acting through the INFLA-score. Metabolomic younger normal weight was set as the reference group. Age, sex, ethnicity, Townsend deprivation index, educational attainment, physical activity, healthy diet, sleep duration, smoking, drinking, and longevity genetic risk scores are the variables included in the models for the mediators and for the outcomes. The asterisk (*) indicates a significant association by two-tailed statistical tests. Abbreviations: CVD, cardiovascular disease; COPD, chronic obstructive pulmonary disease | | | | |


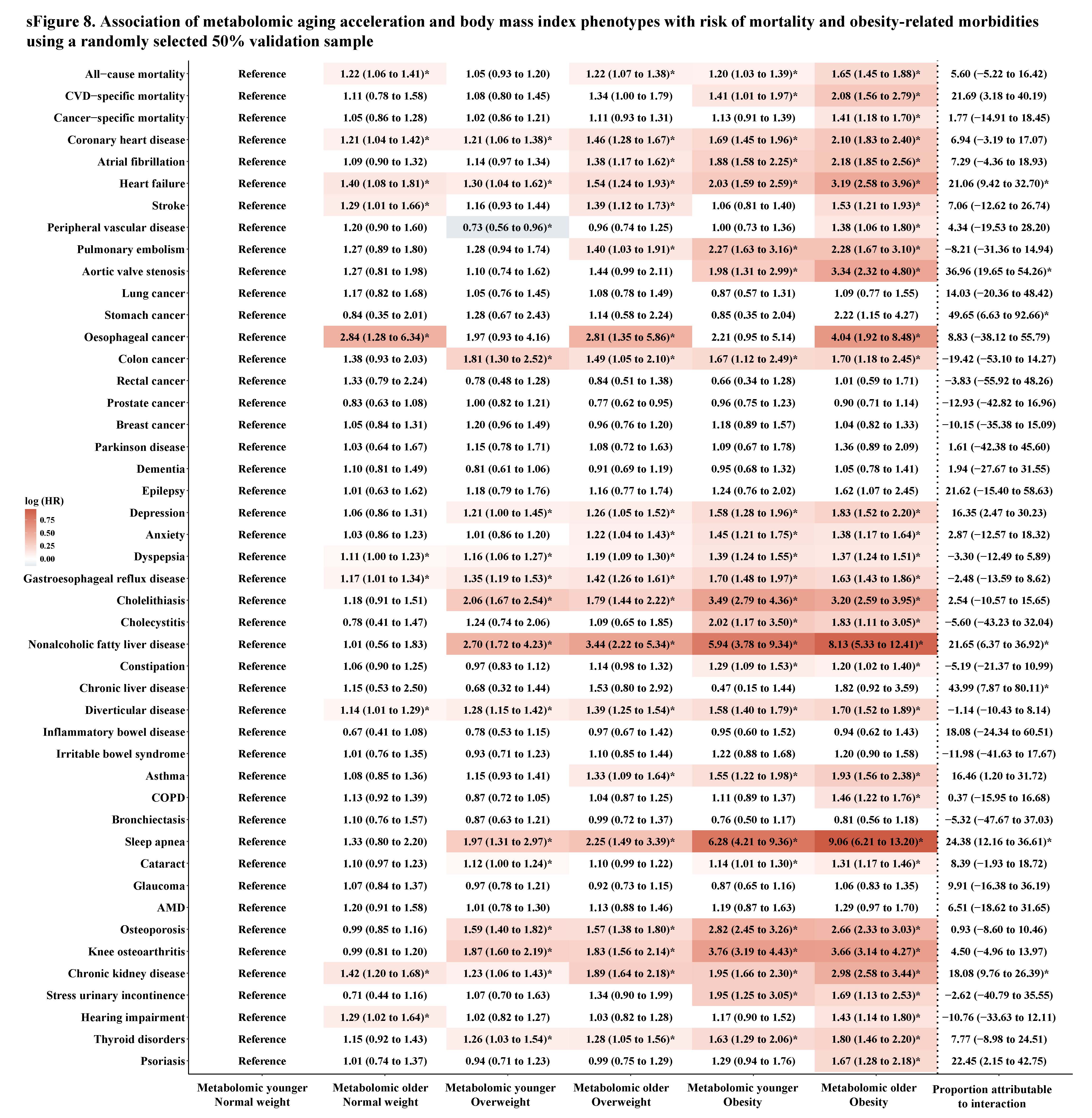


**sFigure 8. Association of metabolomic aging acceleration and body mass index phenotypes with risk of mortality and obesity-related morbidities using a randomly selected 50% validation sample**

The cox proportional hazard regression was used to estimate the association of metabolomic aging acceleration and body mass index phenotypes with risk of mortality and obesity-related morbidities using a randomly selected 50% validation sample, adjusting for age, sex, ethnicity, Townsend deprivation index, educational attainment, physical activity, healthy diet, sleep duration, smoking, drinking, and longevity genetic risk scores. Metabolomic younger normal weight was set as the reference group. Prostate cancer was analyzed only in males. Breast cancer was analyzed only in females. Proportion attributable due to interaction and corresponding 95% confidence intervals was used as the measure of additive interaction between the metabolomic aging acceleration (younger vs older) and obesity status (normal weight vs obesity), and the additive interaction was statistically significant when its confidence interval did not include 0. The asterisk (*) indicates a significant association through two-sided statistical tests.

Abbreviations: HR, hazard ratio; CVD, cardiovascular disease; COPD, chronic obstructive pulmonary disease; AMD, age-related macular degeneration.

| **sTable 5. ICD codes for diseases of interest** | | | |
| --- | --- | --- | --- |
| Long term condition grouping | Conditions included as reported by participants | ICD10 code | ICD9 code |
| 1. Coronary Heart Disease | Angina pectoris | I20 | 413 |
|  | Acute myocardial infarction | I21 | 410 |
|  | Subsequent myocardial infarction | I22 | 411 |
|  | Certain current complications following acute myocardial infarction | I23 | 412 |
|  | Other acute ischaemic heart diseases | I24 | 414 |
|  | Chronic ischaemic heart disease | I25 |  |
| 2. Atrial Fibrillation | Atrial fibrillation and flutter | I48 | 427 |
|  | Paroxysmal atrial fibrillation | I480 |  |
|  | Persistent atrial fibrillation | I481 |  |
|  | Chronic atrial fibrillation | I482 |  |
|  | Atrial fibrillation and atrial flutter, unspecified | I489 |  |
| 3. Heart failure | Cardiomyopathy | I42 | 425 |
|  | Heart failure | I50 | 428 |
| 4. Stroke/Transient Ischaemic Attack (TIA) | Stroke, not specified as haemorrhage or infarction | I64 | 438 |
|  | Occlusion and stenosis of precerebral arteries, not resulting in cerebral infarction | I65 | 435 |
|  | Subarachnoid haemorrhage | I60 | 430 |
|  | Intracerebral haemorrhage | I61 | 431 |
|  | Other nontraumatic intracranial haemorrhage | I62 | 432 |
|  | Occlusion and stenosis of cerebral arteries, not resulting in cerebral infarction | I66 | 433 |
|  | Cerebral infarction | I63 | 434 |
|  | Acute but ill-defined cerebrovascular disease |  | 436 |
|  | Other and ill-defined cerebrovascular disease |  | 437 |
| 5. Peripheral vascular disease | Other aneurysm | I72 | 440 |
|  | Other peripheral vascular diseases | I73 | 443 |
|  | Arterial embolism and thrombosis |  | 444 |
| 6. Pulmonary embolism | Pulmonary embolism with mention of acute cor pulmonale | I260 | 4151 |
|  | Pulmonary embolism without mention of acute cor pulmonale | I269 |  |
| 7. Aortic valve stenosis | Aortic (valve) stenosis | I350 |  |
|  | Aortic (valve) stenosis with insufficiency | I352 |  |
| 8. Lung Cancer | Malignant neoplasm of bronchus and lung | C34 | 162 |
| 9. Stomach cancer | Malignant neoplasm of stomach | C16 | 151 |
| 10. Oesophageal cancer | Malignant neoplasm of oesophagus | C15 | 150 |
| 11. Colon cancer | Malignant neoplasm of colon | C18 | 153 |
| 12. Rectal cancer | Malignant neoplasm of rectum | C20 | 154 |
| 13. prostate cancer | Malignant neoplasm of prostate | C61 | 185 |
| 14. Breast cancer | Malignant neoplasm of breast | C50 | 174 |
| 15. Parkinson’s disease | Parkinson's disease | G20 | 332 |
|  | Secondary Parkinsonism | G21 | 3321 |
|  | Parkinsonism in diseases classified elsewhere | G22 | 333 |
|  | Other degenerative diseases of basal ganglia | G23 |  |
|  | Extrapyramidal and movement disorder, unspecified | G259 |  |
|  | Extrapyramidal and movement disorders in diseases classified elsewhere | G26 |  |
|  | Multisystem degeneration | G903 |  |
| 16. Dementia | Dementia in Alzheimer's disease with early onset | F00 |  |
|  | Alzheimer's disease | G30 | 3310 |
|  | Vascular dementia | F01 | 2904 |
|  | Progressive vascular leukoencephalopathy | I673 |  |
|  | Dementia in Pick's disease | F020 | 3311 |
|  | Circumscribed brain atrophy | G310 |  |
|  | Creutzfeldt-Jakob disease | A810 | 3315 |
|  | Dementia in other diseases classified elsewhere | F02 | 2941 |
|  | Unspecified dementia | F03 |  |
|  | Delirium superimposed on dementia | F051 |  |
|  | Amnesic syndrome | F106 |  |
|  | Senile degeneration of brain, not elsewhere classified | G311 | 3312 |
|  | Other specified degenerative diseases of nervous system | G318 |  |
| 17. Epilepsy | Epilepsy | G40 | 345 |
| 18. Depression | Depressive episode | F32 | 2962 |
|  | Recurrent depressive disorder | F33 | 2963 |
|  | Dysthymia | F341 | 3004 |
|  | Other recurrent mood [affective] disorders | F381 | 311 |
|  | Postschizophrenic depression | F204 |  |
| 19. Anxiety | Phobic anxiety disorders | F40 | 3000 |
|  | Other anxiety disorders | F41 | 3002 |
|  | Reaction to severe stress, and adjustment disorders | F43 | 3009 |
|  | Posttraumatic stress disorder | F431 |  |
|  | Obsessive-compulsive disorder | F42 | 3003 |
|  | Stress, not elsewhere classified | Z733 | 308 |
|  | Disorders of initiating and maintaining sleep [insomnias] | G470 | 7805 |
|  | Mental disorder, not otherwise specified | F99 |  |
| 20. Dyspepsia | Gastro-oesophageal reflux disease | K21 | 53081 |
|  | Gastro-oesophageal reflux disease with oesophagitis | K210 | 5368 |
|  | Gastro-oesophageal reflux disease without oesophagitis | K219 |  |
|  | Oesophagitis | K20 |  |
|  | Barrett's oesophagus | K227 |  |
|  | Other specified diseases of oesophagus | K228 |  |
|  | Disease of oesophagus, unspecified | K229 |  |
|  | Disorders of oesophagus in diseases classified elsewhere | K23 |  |
|  | Gastric ulcer | K25 |  |
|  | Gastritis and duodenitis | K29 |  |
|  | Duodenal ulcer | K26 |  |
|  | Dyspepsia | K30 |  |
|  | Congenital hiatus hernia | Q401 |  |
|  | Helicobacter pylori [H.pylori] as the cause of diseases classified to other chapters | B980 |  |
| 21. Gastroesophageal reflux disease | Gastro-oesophageal reflux disease with oesophagitis | K210 |  |
|  | Gastro-oesophageal reflux disease without oesophagitis | K219 |  |
| 22. Cholelithiasis | Calculus of gallbladder with acute cholecystitis | K800 |  |
|  | Calculus of gallbladder with other cholecystitis | K801 |  |
|  | Calculus of gallbladder without cholecystitis | K802 |  |
|  | Calculus of bile duct with cholangitis | K803 |  |
|  | Calculus of bile duct with acute and chronic cholangitis without obstruction | K8036 |  |
|  | K80.4 Calculus of bile duct with cholecystitis | K804 |  |
|  | Calculus of bile duct without cholangitis or cholecystitis | K805 |  |
|  | Other cholelithiasis | K808 |  |
| 23. Cholecystitis | Acute cholecystitis | IK810 |  |
|  | Chronic cholecystitis | K811 |  |
|  | Other cholecystitis | K818 |  |
|  | Cholecystitis, unspecified | K819 |  |
| 24. Nonalcoholic fatty liver disease | Fatty (change of) liver, not elsewhere classified | K760 |  |
|  | Other chronic nonalcoholic liver disease |  | 5718 |
| 25. Constipation | Constipation | K590 | 5640 |
| 26. Chronic liver disease | Oesophageal varices | I85 | 571 |
|  | Toxoplasma hepatitis | B581 |  |
|  | Alcoholic hepatitis | K701 |  |
|  | Toxic liver disease with acute hepatitis | K712 |  |
|  | Toxic liver disease with chronic persistent hepatitis | K713 |  |
|  | Toxic liver disease with chronic lobular hepatitis | K714 |  |
|  | Toxic liver disease with chronic active hepatitis | K715 |  |
|  | Toxic liver disease with hepatitis, not elsewhere classified | K716 |  |
|  | Fibrosis and cirrhosis of liver | K74 |  |
|  | Primary biliary cirrhosis | K743 |  |
| 27. Diverticular disease | Diverticular disease of intestine | K57 | 562 |
| 28. Inflammatory Bowel Disease | Crohn's disease [regional enteritis] | K50 |  |
|  | Ulcerative colitis | K51 |  |
| 29. Irritable bowel syndrome | Irritable bowel syndrome | K58 | 5641 |
| 30. Asthma | Asthma | J45 | 493 |
|  | Predominantly allergic asthma | J450 |  |
|  | Nonallergic asthma | J451 |  |
|  | Mixed asthma | J458 |  |
|  | Asthma, unspecified | J459 |  |
| 31. Chronic obstructive pulmonary disease (COPD) | Simple and mucopurulent chronic bronchitis | J41 | 491 |
|  | Unspecified chronic bronchitis | J42 |  |
|  | Emphysema | J43 | 492 |
|  | Other chronic obstructive pulmonary disease | J44 | 494 |
| 32. Bronchiectasis | Bronchiectasis | J47 | 494 |
| 33. Sleep apnea | Sleep apnoea | G473 |  |
| 34. Cataract | Senile cataract | H25 | 366 |
|  | Other cataract | H26 |  |
|  | Cataract and other disorders of lens in diseases classified elsewhere | H28 |  |
| 35. Glaucoma | Glaucoma | H40 | 365 |
| 36. AMD | Degeneration of macula and posterior pole | H353 | 3625 |
| 37. Osteoporosis | Polyarthrosis | M15 | 7330 |
|  | Primary generalised (osteo)arthrosis | M150 |  |
|  | Primary generalized (osteo)arthrosis, Multiple sites | M1500 |  |
|  | Heberden's nodes (with arthropathy) | M151 |  |
|  | Coxarthrosis [arthrosis of hip] | M16 |  |
|  | Gonarthrosis [arthrosis of knee] | M17 |  |
| 38. Knee osteoarthritis | Primary gonarthrosis, bilateral | M170 |  |
|  | Other primary gonarthrosis | M171 |  |
|  | Gonarthrosis, unspecified | M179 |  |
|  | Localised, primary osteoarthrosis and allied disorders (lower leg) |  | 71516 |
|  | Unspec. localised osteoarthrosis/allied dis. (lower leg) |  | 71536 |
| 39. Chronic kidney disease | Polycystic kidney, infantile type | Q611 | 75315 |
|  | Polycystic kidney, adult type | Q612 |  |
|  | Polycystic kidney, unspecified | Q613 |  |
|  | Acute renal failure | N17 | 584 |
|  | Chronic renal failure | N18 | 585 |
|  | Unspecified renal failure | N19 | 586 |
|  | Renal complications | E112 | 587 |
|  | Other | N028 | 588 |
| 40. Stress urinary incontinence | Stress incontinence | N393 | 6256 |
| 41. Hearing impairment | Conductive and sensorineural hearing loss | H90 | 389 |
|  | Other and unspecified hearing loss | H91 |  |
| 42. Thyroid disorders | Thyrotoxicosis [hyperthyroidism] | E05 | 241 |
|  | Other hypothyroidism | E03 | 242 |
|  | Other non-toxic goitre | E04 | 243 |
|  | Iodine-deficiency-related thyroid disorders and allied conditions | E01 | 244 |
|  | Thyroiditis | E06 | 245 |
|  | Other disorders of thyroid | E07 | 246 |
|  | Subclinical iodine-deficiency hypothyroidism | E02 |  |
| 43. Psoriasis | Atopic dermatitis | L20 | 696 |
|  | Seborrhoeic dermatitis | L21 | 692 |
|  | Diaper [napkin] dermatitis | L22 |  |
|  | Allergic contact dermatitis | L23 |  |
|  | Irritant contact dermatitis | L24 |  |
|  | Unspecified contact dermatitis | L25 |  |
|  | Exfoliative dermatitis | L26 |  |
|  | Dermatitis due to substances taken internally | L27 |  |
|  | Other dermatitis | L30 |  |
|  | Psoriasis | L40 |  |
|  | Parapsoriasis | L41 |  |

| **sTable 6. Self-reported codes for diseases of interest** | | |
| --- | --- | --- |
| **Long term condition grouping** | **Conditions included as reported by participants** | **Field Code** |
| 1. Coronary heart disease | Heart attack/Myocardial Infarction | 1075 |
|  | Angina | 1074 |
| 2. Atrial Fibrillation | Atrial Fibrillation | 1471 |
| 3. Heart failure | Cardiomyopathy | 1240 |
|  | Hypertrophic cardiomyopathy | 1588 |
|  | Heart failure/pulmonary oedema | 1076 |
| 4. Stroke/Transient Ischaemic Attack (TIA) | Stroke | 1081 |
|  | TIA | 1082 |
|  | Subarachnoid haemorrhage | 1083 |
|  | Brain haemorrhage | 1086 |
|  | Ischaemic stroke | 1583 |
| 5. Peripheral vascular disease | Peripheral vascular disease | 1067 |
|  | Leg claudication/intermittent claudication | 1087 |
| 6. Pulmonary embolism | pulmonary embolism +/- dvt | 1093 |
| 7. Aortic valve stenosis | aortic stenosis | 1490 |
| 8. Lung Cancer | Lung Cancer | 1001 |
| 9. Stomach cancer | Stomach cancer | 1018 |
| 10. Oesophageal cancer | Oesophageal cancer | 1017 |
| 11. Colon cancer | Colon cancer | 1022 |
| 12. Rectal cancer | Rectal_cancer | 1023 |
| 13. prostate cancer | Prostate cancer | 1044 |
| 14. Breast cancer | Breast cancer | 1002 |
| 15. Parkinson’s disease | Parkinson’s disease | 1262 |
| 16. Dementia | Dementia/alzheimers/cognitive impairment | 1263 |
| 17. Epilepsy | Epilepsy | 1264 |
| 18. Depression | Depression | 1286 |
|  | Postnatal Depression | 1531 |
| 19. Anxiety | Anxiety/panic attacks | 1287 |
|  | Nervous breakdown | 1288 |
|  | Post-traumatic stress disorder | 1469 |
|  | Obsessive compulsive disorder | 1615 |
|  | Stress | 1614 |
|  | Insomnia | 1616 |
|  | Psychological/psychiatric problem | 1243 |
| 20. Dyspepsia | Gastro-oesophageal reflux (GORD)/gastric reflux | 1138 |
|  | Oesophagitis /Barrett's oesophagus | 1139 |
|  | Gastric stomach ulcers | 1142 |
|  | Gastric erosions/gastritis | 1143 |
|  | Duodenal ulcer | 1457 |
|  | Dyspepsia/indigestion | 1510 |
|  | Hiatus hernia | 1474 |
|  | Helicobacter pylori | 1442 |
| 21. Gastroesophageal reflux disease | gastro-oesophageal reflux (gord) / gastric reflux | 1138 |
| 22. Cholelithiasis | cholelithiasis/gall stones | 1162 |
| 23. Cholecystitis | cholecystitis | 1163 |
| 24. Nonalcoholic fatty liver disease |  | NA |
| 25. Constipation | Constipation | 1599 |
| 26. Chronic liver disease | Oesophageal varices | 1141 |
|  | Non infective hepatitis | 1157 |
|  | Liver failure/cirrhosis | 1158 |
|  | Primary biliary cirrhosis | 1506 |
| 27. Diverticular disease | Diverticular disease | 1458 |
|  | Diverticulitis | 1458 |
| 28. Inflammatory bowel disease | Inflammatory Bowel Disease | 1461 |
|  | Crohn’s disease | 1462 |
|  | Ulcerative colitis | 1463 |
| 29. Irritable bowel syndrome | Irritable bowel syndrome | 1154 |
| 30. Asthma | Asthma | 1111 |
| 31. Chronic Obstructive Pulmonary Disease (COPD) | COPD/chronic obstructive airways disease | 1112 |
|  | Emphysema/chronic bronchitis | 1113 |
|  | Emphysema | 1472 |
| 32. Bronchiectasis | Bronchiectasis | 1114 |
| 33. Sleep apnea | sleep apnoea | 1123 |
| 34. Cataract | Cataract | 1278 |
| 35. Glaucoma | Glaucoma | 1277 |
| 36. AMD | Macular degeneration | 1528 |
| 37. Osteoporosis | Osteoporosis | 1465 |
| 38. Knee osteoarthritis |  | NA |
| 39. Chronic kidney disease | Polycystic kidney | 1427 |
|  | Diabetic nephropathy | 1607 |
|  | Renal/kidney failure | 1192 |
|  | Renal failure requiring dialysis | 1193 |
|  | Renal failure not requiring dialysis | 1194 |
|  | Kidney nephropathy | 1519 |
|  | Immunoglobulin A (IgA) nephropathy | 1520 |
| 40. Stress urinary incontinence |  | NA |
| 41. Hearing impairment | Hearing aid | 2247 |
|  | Difficult to follow a conversation | 2257 |
| 42. Thyroid disorders | Thyroid problem (not cancer) | 1224 |
|  | Hyperthyroidism/thyrotoxicosis | 1225 |
|  | Hypothyroidism/myxoedema | 1226 |
|  | Grave’s disease | 1522 |
|  | Thyroid goitre | 1610 |
|  | Thyroiditis | 1428 |
| 43. Psoriasis | Eczema/dermatitis | 1452 |
|  | Psoriasis | 1453 |
